# The Past, Present, and Future of Non-Viral CAR T Cells

**DOI:** 10.3389/fimmu.2022.867013

**Published:** 2022-06-09

**Authors:** Alex Moretti, Marianna Ponzo, Charles A. Nicolette, Irina Y. Tcherepanova, Andrea Biondi, Chiara F. Magnani

**Affiliations:** ^1^ Tettamanti Research Center, Department of Pediatrics, University of Milano-Bicocca/Fondazione Monza e Brianza per il Bambino e la sua Mamma (MBBM), Monza, Italy; ^2^ CoImmune Inc., Durham, NC, United States; ^3^ Department of Pediatrics, University of Milano - Bicocca, Milan, Italy; ^4^ Clinica Pediatrica, University of Milano - Bicocca/Fondazione MBBM, Monza, Italy; ^5^ Department of Medical Oncology and Hematology, University Hospital Zurich and University of Zurich, Zurich, Switzerland

**Keywords:** non-viral vectors, chimeric antigen receptor (CAR T), gene therapy, immunotherapy, adoptive cell transfer, cancer therapy, transposons, mRNA

## Abstract

Adoptive transfer of chimeric antigen receptor (CAR) T lymphocytes is a powerful technology that has revolutionized the way we conceive immunotherapy. The impressive clinical results of complete and prolonged response in refractory and relapsed diseases have shifted the landscape of treatment for hematological malignancies, particularly those of lymphoid origin, and opens up new possibilities for the treatment of solid neoplasms. However, the widening use of cell therapy is hampered by the accessibility to viral vectors that are commonly used for T cell transfection. In the era of messenger RNA (mRNA) vaccines and CRISPR/Cas (clustered regularly interspaced short palindromic repeat–CRISPR-associated) precise genome editing, novel and virus-free methods for T cell engineering are emerging as a more versatile, flexible, and sustainable alternative for next-generation CAR T cell manufacturing. Here, we discuss how the use of non-viral vectors can address some of the limitations of the viral methods of gene transfer and allow us to deliver genetic information in a stable, effective and straightforward manner. In particular, we address the main transposon systems such as Sleeping Beauty (SB) and piggyBac (PB), the utilization of mRNA, and innovative approaches of nanotechnology like Lipid-based and Polymer-based DNA nanocarriers and nanovectors. We also describe the most relevant preclinical data that have recently led to the use of non-viral gene therapy in emerging clinical trials, and the related safety and efficacy aspects. We will also provide practical considerations for future trials to enable successful and safe cell therapy with non-viral methods for CAR T cell generation.

## 1 Introduction

### 1.1 The Rise of CAR T Immunotherapy in Hematological Malignancies

Chimeric antigen receptor (CAR) T cell therapy represents a revolutionary therapeutic reality. To unleash T cells against cancer, an artificial receptor has been generated fusing the antigen-binding domain of a monoclonal antibody with a T-cell receptor (TCR)-derived signaling domain, including costimulatory components (1, 2). CAR-mediated recognition of a tumor-associated antigen triggers the activation of engineered T cells that consequently exert a response, characterized by potent cytotoxicity, cytokine secretion, and proliferation. The possibility of combining T cell lymphocyte effector functions with antibody specificity in a single component is appealing because it allows for a T cell-mediated immune response against the tumor in a major histocompatibility complex (MHC)-unrestricted manner. This strategy eliminates the need of designing different receptors according to the Human Leukocyte Antigen (HLA) haplotypes, as in the case of tumor specific TCR gene transfer. This type of immunotherapy is a multi-step process. Immune cells, typically of autologous origin, are collected, modified in specialized laboratories, and then infused into the patient undergoing lymphodepleting therapy to increase engraftment. The early concept formulated more than 30 years ago went through an extensive series of costimulatory design optimizations, which coupled the CD3-ζ domain with CD28 or 4-1BB costimulation, leading to impressive clinical results in patients with high-risk hematological malignancies (3–8).

**Figure 1:**
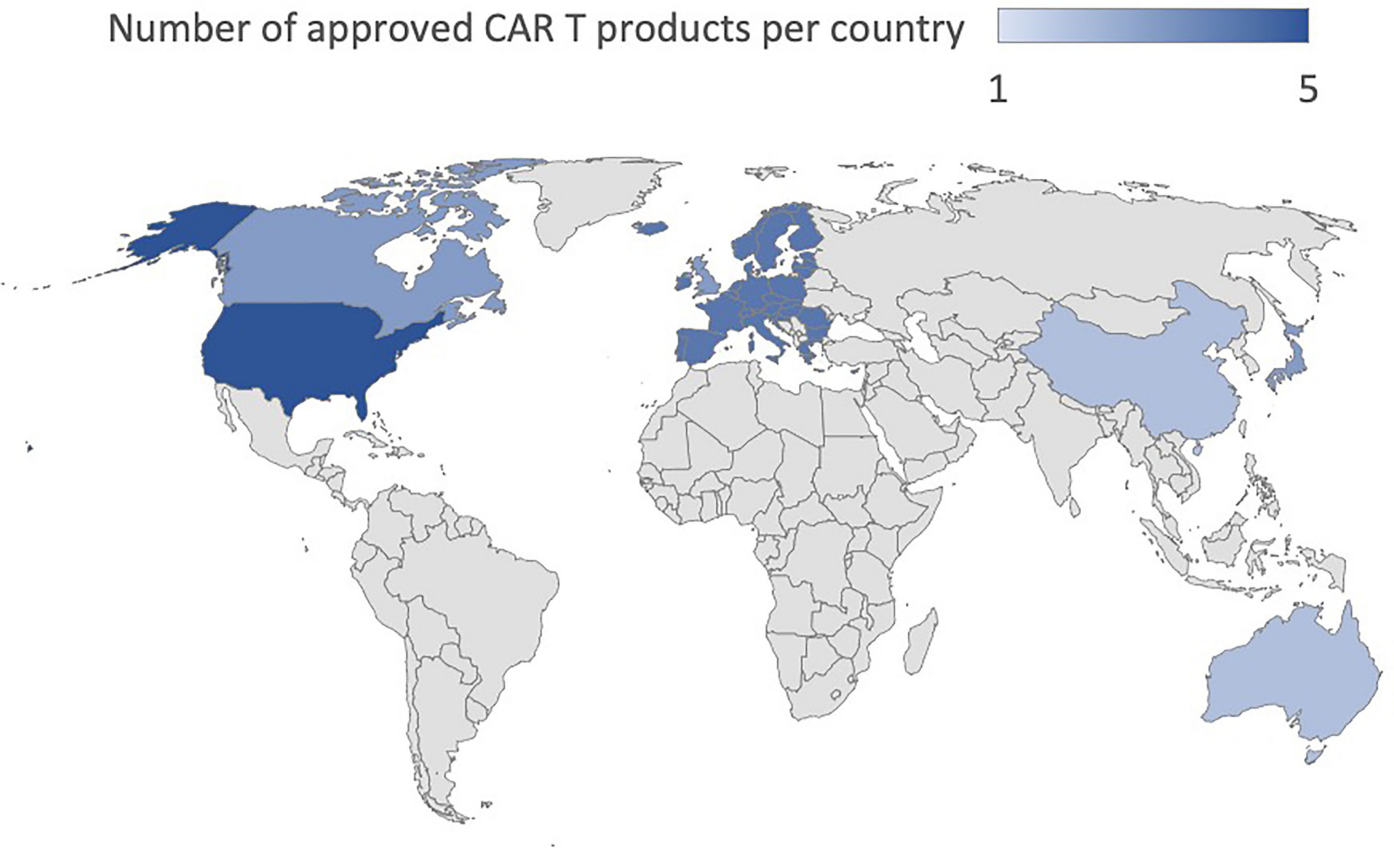
Worldwide approval status of CAR T cell drugs.

To date, the clinical application that has determined the success of CAR T cells has been conducted mainly targeting the CD19 and CD22 molecules in B cell-Acute Lymphoblastic Leukemia (B-ALL) and B cell lymphoma (9–12) and against B-Cell Maturation Antigen (BCMA) for multiple myeloma (13, 14). In B-ALL, adoptive immunotherapy with CAR T cells achieved more than 80% complete response (CR) in the early stages of treatment and a sustained response through the establishment of immunological memory with 12-month event-free survival rates of 50% (3, 15). In diffuse large B-cell lymphoma (DLBCL), the CR was between 40 and 60% in multiple studies with different CAR T cell products while 12-months progression-free survival (PFS) was 40% (10, 11, 16, 17). The results in Mantle Cell Lymphoma and Relapsed/Refractory (R/R) Follicular Lymphoma are even more encouraging with a CR of 67% and 80%, and a PFS of 61% and 74%, respectively (18, 19). Finally, in Multiple Myeloma (MM), the CR is 33% while the PFS is 8.8 months (20). In light of these data (21), the U.S Food and Drug Administration (FDA) has approved five CAR T cell therapies, Abecma (idecabtagene vicleucel), Breyanzi (lisocabtagene maraleucel), Kymriah (tisagenlecleucel), Tecartus (brexucabtagene autoleucel), Yescarta (axicabtagene ciloleucel) as of January 2022. All of them are also authorized in Europe. Besides these five, the FDA recently approved ciltacabtagene autoleucel (CARVYKTI, cilta-cel, Janssen and Johnson & Johnson), a CAR T product direct against BCMA. Furthermore, China’s National Medical Products Administration (NMPA) recently approved the autologous anti-CD19 CAR T cell product, relmacabtagene autoleucel, that was established based on a process platform of Juno Therapeutics. Progress has been made to implement CAR T cell therapies in Australia, China, Japan, Switzerland, Singapore and Canada and the approval status worldwide is summarized in **Table 1** and **Figure 1**. Kymriah and Yescarta have been commercially available since 2017 and 2018, respectively, and have been infused into nearly half a million patients worldwide.

**Table 1:** Commercial CAR T products and their indication and availability worldwide.

Active substance	Name	Indications	Manufacturer	Approvals	Target	Costimulatory domain
tisagenlecleucel	Kymriah	Pediatric and young adult R/R acute lymphoblastic leukemia; Adult R/R DLBCL; R/R follicular lymphoma	Novartis	FDA, EMA, Health Canada, Swissmedic, Japan’s MHLW, Singapore’s HSA, Australian TGA, UK’s NICE	CD19	CD137
axicabtagene ciloleucel	Yescarta	R/R large B-cell lymphoma (DLBCL, PMBCL, high grade B-cell lymphoma, DLBCL arising from FL)	Kite Pharma and Gilead	FDA, EMA, Health Canada, Swissmedic, Japan’s MHLW, China’s NMPA, Australian TGA, UK’s NICE	CD19	CD28
brexucabtagene autoleucel	Tecartus	Mantle cell lymphoma; Adult lymphoblastic leukemia	Kite Pharma and Gilead	FDA, EMA, Swissmedic, UK’s NICE, Health Canada	CD19	CD28
lisocabtagene maraleuecel	Breyanzi	R/R large B-cell lymphoma	BMS and Juno Therapeutics	FDA, Japan’s MHLW, EMA	CD19	CD137
idecabtagene vicleucel	Abecma	Multiple myeloma	BMS and Bluebird Bio	FDA, EMA, Health Canada, Swissmedic, Japan	BCMA	CD137
ciltacabtagene autoleucel	CARVYKTI	Multiple myeloma	Janssen and Johnson & Johnson	FDA	BCMA	CD137
relmacabtagene autoleucel	Carteyva	R/R large B-cell lymphoma	JW Therapeutics	China’s NMPA	CD19	CD137

MHLW, Ministry of Health, Labor and Welfare; HAS, Health Sciences Authority; TGA, Therapeutic Goods Administration; NMPA, National Medical Products Administration; NICE The National Institute for Health and Care Excellence.

### 1.2 Need for a More Flexible System to Allow Future CAR T Cell Engineering

Having shown such high therapeutic efficacy in hematological malignancies, the field is moving surprisingly fast, facing new challenges, and approaching other kinds of applications. Despite the high CR rate in hematological malignancies, patients with a high tumor burden and characterized by a history of multiple prior lines of therapy often do not respond. In some cases, the achieved responses do not last long. Relapses are mainly associated with loss of functional CAR T cells or the appearance of relapses in which the antigen recognized by the CAR has a decreased expression or is completely absent, as in the case of CD19-negative relapses. For solid tumors, treatment with CAR T cells have not yet proven to be efficacious and it remains a challenge as of today. Few antigens with restricted expression to solid cancer and non-vital organs have been identified so far. Homing to the tumor is a critical aspect because T cells must migrate from the bloodstream through the endothelial cells that make up the tumor vasculature. In addition, the tumor microenvironment often has an immunosuppressive and hypoxic environment that impacts on T cell persistence by inducing a hypofunctional state. Unfortunately, clinical studies in solid tumors demonstrate a severely limited response. With this in mind, it is increasingly becoming urgent to combine CAR weapons with multiple targeting options and different functionalities, i.e., *de novo* cytokine production, activating signaling molecules and pro-inflammatory ligands, checkpoint blockages, increased trafficking with chemokines, receptors and extracellular matrix degrading enzyme, safety switches. Some of the issues associated to the current CAR T cell design that we need to face in the future are summarized in (22).

Addressing such challenges requires the development of approaches that move beyond single-target immunotherapy towards a building-block concept à la Lego or Minecraft, the popular video game that allows for endless combination opportunity. T cells can be modified to express CARs with different specificities and can therefore be equipped to improved efficacy, safety, and applicability. So far, the way most CAR T cell therapies approved or investigated in clinical trials are produced is utilizing viral vectors, particularly gammaretroviral and lentiviral vectors. Viral vectors are standardized systems with efficient gene transfer and a long‐term history of application that demonstrates safety in the context of adoptive T cell therapy (23). However, the ability of viral vectors to transduce long gene cassettes is constrained by the capsid dimension. Viral capsids are about 100 nm in diameter and often cannot fit more than 8-9 kb (24). The use of two separate vectors for delivery of two different transgenes is often not efficient. Furthermore, viral production for the clinical application is a process that generally takes two-three weeks and is performed under good manufacturing practice (GMP) conditions in biosafety level 2 (BSL2) facilities, needing trained staff resources. The resulting high costs, limited number of available manufacturing facilities globally, and lot size limitations complicate their accessibility. This complexity along with the need for personalized treatment ultimately impacts the final price of CAR T product, which is particularly high and can reach up to $475,000 per person to which must be added the cost of hospitalization, and follow-up visits. Currently, CAR T therapies are often recommended for late-stage patients who have exhausted all other treatment options. Given recent data supporting the advancement of CAR T cell therapy to earlier lines of treatment (25, 26), it appears to be increasingly important to implement reductions in current spending.

For the future to come, genetic engineering technologies must address issues such as logistical complexities impacting on costs and availability, cargo limitations, and flexibility. We therefore need to take measures to mitigate these challenges and start using more versatile and flexible technologies to make CAR T cells capable of migrating to the tumor site, recognizing heterogeneous tumors, and surviving in hostile environments. To support the adoption of future CAR T cell therapies, non-viral vectors have been proposed, validated preclinically in their ability to generate functional CAR T cells, and more recently applied in pioneer clinical trials. Non-viral gene transfer allows for an easier manufacturing process with lower costs of goods and rapid availability and may have less constraint on cargo capacity.

### 1.3 Stable Gene Transfer: Viral Transduction vs. Non-Viral Transfection

Gene therapy is an advanced medicine application in which the delivery of genetic material into cells is exploited to confer additional or restore impaired features to treat patients with a wide range of diseases, including genetic disorders, cancer, infectious and immunologic diseases, resulting in long-term therapeutic effects. Gene transfer can be classified as stable or transient depending on whether the genetic material is integrated into the host cell genome. To achieve stable gene transfer, integration of an expression cassette consisting of promoter, leader, transgene and transcriptional termination and polyadenylation sequences is needed. When there is integration into the genome, the transgene is stably expressed, and expression of the inserted gene persists in daughter cells resulting from cell division. In contrast, in the absence of integration, as for mRNA and plasmid vectors, expression will be lost as cells divide. Adoptive cell therapy with CAR T cells utilize *ex vivo* gene therapy that predominantly uses stable gene transfer. Gene transfer can be achieved through the process of viral transduction that utilizes the inherent ability of viruses and viral vectors to introduce genetic material into a variety of cell types. Alternatively, introduction of naked nucleic acids, including supercoiled DNA, messenger RNA (mRNA), small interfering RNA (siRNA), and guide RNA (gRNA), can be achieved by non-viral transfection. Transfection relies on the formation of transient pores in the cell membrane or, alternatively, endocytosis through the use of different chemical or physical techniques, such as electroporation, liposomes, and nanoparticles.

#### 1.3.1 Viral Transduction

In the case of retroviral vectors, transduction requires the formation of infectious particles containing the transfer plasmid encoding the transgene flanked by Long Terminal Repeat (LTRs) and including the ψ (psi) encapsidation signal. Generally, infectious particles are generated by introducing the necessary viral sequences, i.e. *gal-pol, rev, env* coding sequences into the producer cell line by means of separated plasmids. Separation of the sequences required for virus formation allows the generation of a replication-deficient virus that is capable of infecting mammalian cells and integrate the genetic materials into the cellular genome but does not retain the natural ability to generate new viruses. Integration of DNA into the genome allows stable transduction of the T cell clone and its lineage, leading to long-term expression of the transgene in cells capable of long-term survival, and thus making CAR T cells living drugs.

The mechanism of integration of the cassette into the genome relies on the action of reverse transcriptase and integrase, encoded by the *pol* gene. Gammaretroviral vectors derived from Moloney murine leukemia virus (MLV) vectors integrate preferentially near transcription start sites (TSS) and in transcriptional regulatory regions, whereas Human Immunodeficiency Virus (HIV)-derived lentiviral vectors have a bias towards transcriptionally active regions (27–29). Integration of a transgene into the genome carries with it the risk of insertional oncogenesis, which is closely related to the propensity of each vector for a particular integration profile. In the case of gammaretroviral vectors there is thus a higher likelihood of inducing aberrant gene expression, which can result in the activation of oncogenes, whereas lentiviruses potentially have a greater risk of disrupting gene expression or leading to the expression of gene fragments that could theoretically lead to tumor-suppressor gene inactivation. However, this is particularly relevant in gene therapy applied to hematopoietic stem and progenitor cells (HSPC) (30, 31), while T cells have been considered to have a low susceptibility to transformation. Indeed, long-term safety has been demonstrated after viral transfection (23). No T cell transformation has been observed even in cases of gammaretroviral vector insertion into an oncogene, such as *Cbl*, and destruction of a tumor suppressor gene such as Tet Methylcytosine dioxygenase 2 (*TET2*) by lentiviral vector integration, as has been reported in patients treated with anti-CD19 and anti-CD22 CAR T cells (32, 33).

The two vectors also differ in the mode of infection, which also has practical implications. Gammaretroviral vectors can only infect cells with active cell division, whereas lentiviral vectors are able to transduce non-dividing as well as the dividing cells, but most current protocols activate T cells prior to transduction (34). In addition, lentiviral genomes are more complex than those of gammaretroviruses, making LV production more complicated. Both viral vectors suffer from a number of disadvantages as gene transfer vectors, including i. limited insert size, ii. difficulty in producing high titers of stable vector particles, iii. potential generation of replication competent retroviruses/lentiviruses (RCR/RCL) during production, and iiii. *in vivo* recombination with sequences from other viruses, such as post HIV infection (35, 36). The generation of RCR/RCL *in vitro* or *in vivo* is currently only a theoretical risk, as there have been no cases of recombination in cellular products or in patients treated with *ex vivo* gene therapy to date. Finally, viral vectors have an intrinsic risk of immunogenicity, caused by humoral and cellular immune response towards vector-encoded epitopes, which might limit the efficacy and persistence of transduced cells (37).

#### 1.3.2 Non-Viral Transfection

Stable gene transfer delivery can also be achieved by using the non-viral integrative vectors represented by transposons. In this case, integration is obtained by means of transposase, an enzyme that binds to sequences in the genome called transposons and catalyzes their movement by a cut-and-paste or a replicative transposition mechanism. The existence of mobile sequences in the genome was originally discovered by the Nobel Prize-winning geneticist Barbara McClintock in the 1940s while studying kernel color variability in maize (38). The repositioning of genes encoding for pigments resulted in a variety of coloration patterns. The “jumping genes” in maize were then called transposable elements (TE), or transposons, and we now know that they are quite abundant in the genome, constituting more than 80% of the maize genome and about 40% of the human’s one, meaning that around 40% of the human genome has undergone the process of transposition over the course of human evolution (39). Transposition is known to cause genetic diversity and adaptability, such as color change in maize or antibiotic resistance in bacteria. This is likely the reason why genes encoding for transposases are widely distributed in the genome of most organisms (40).This class of genes belongs to the superfamily of polynucleotidyl transferases that comprises RNase H, Recombination-activating gene (RAG) proteins, and retroviral integrases. Indeed, RAG enzymes have been proposed to originate from TEs and have a pretty similar mechanistic features (41), that allow them to alter gene structure as in V(D)J rearrangements.

TEs are divided into two classes of TE, retrotransposons and DNA transposons. Retrotransposons move through a copy-and-paste mechanism using an RNA intermediate, represent the most frequent class of transposons in the human genome, and comprise Long Terminal Repeat (LTR) transposons, long interspersed nuclear elements (LINEs), and short interspersed nuclear elements (SINEs) (42). DNA transposable elements move through a DNA intermediate *via* a cut-and-paste mechanism and are the ones used in gene transfer applications. Most DNA transposon families have an element encoding a transposase gene flanked by inverted terminal repeats (ITRs). Transposase recognizes and binds elements incorporated into ITRs, catalyzes the excision of the transposon element from its original position, and integrates it into another position in the genome. The DNA sequence is inserted without the need for sequence homology. Transposon-based vector systems have been generated by splitting the transposase and the ITRs into two components, so that the transgene cassette lies between the two ITRs in a transposon vector. Throughout the next section, we will focus on the different transposons available for clinical applications, with an emphasis on the most widely used transposon systems, Sleeping Beauty (SB) and piggyBac (PB). Transposase is delivered in a ‘trans’ configuration to better control the system and avoid residual expression, which could then potentially lead to remobilization of the transposon into other genomic compartments which is currently the most prominent safety concern of this type of vector. One of the possibilities to deliver transposon and transposase to generate CAR T cells is through the use of a dual plasmid system, one for the gene of interest and the other for the enzyme, by electroporation of primary T cells, but has some limitations that can be solved by using mRNA and DNA vectors with decreased size compared to conventional plasmids. Anyway, these two-component vector systems are less complex than viral vectors and relies on relatively low costs of goods. Plasmids can be produced in very large quantities, so that the estimated costs are 5 to 10 times lower than the viral process (43). Compared to other vectors, they have a larger cargo size which is particularly relevant for future multi-targeting applications. The integration profile of transposon vectors depends on the transposase used, with some showing a bias towards specific regions, as in viral vectors, while others demonstrate a close-to-random and safer profile with no preference, and we will see how relevant this is in the next subsection. Unlike viral vectors, transfection with transposon systems works well in both pre-activated and resting primary T cells, leading to transgene expression even in naïve cells (44). The ability of transposons to transfect non-dividing and naïve cells might be exploited to increase the persistence of CAR T cells *in vivo*. In contrast to viral systems, which have weak preferences at the site of integration in terms of DNA sequence recognition, transposons recognize a consensus sequence (45).

Non-viral transposon vectors prove versatility, low immunogenicity, and ease of production. However, they are often associated with lower transfection efficiencies than viruses. This may be in part due to the toxicity associated with electroporation in the presence of DNA. Though, there could also be reasons related to the type of gene material, viral material being generally more efficient than plasmid DNA, and the integration pattern itself. Finally, the possibility of transposon vectors interacting with endogenous human DNA and protein sequences is a theoretical safety concern. Fortunately, mammals do not contain transposon DNA sequences sufficiently similar to be cleaved by SB transposase, and there is no human protein sufficiently similar to SB transposase to re-mobilize a SB vector integrated into the genome. Instead, the human *PGBD5* gene, apparently derived from PB transposases, has been shown to encode for a transposase capable of mobilizing insect PB transposons in human cell cultures (46). It remains unclear whether cross-reactions between the endogenous human transposase and the PB transposon vector can occur and undermine the genomic integrity of the transduced cells, raising a potential risk in the context of using this vector for genetic engineering (47).

Non-viral delivery of mRNA allows for transient transfection and is generally achieved by electroporation or nanoparticles (48). Once into the cell and without the need to reach the cell nucleus, the mRNA is translated into the encoded protein that can be stabilized when prolonged expression is needed and is generally lost after 2-4 cell divisions, which is why this technique is particularly suited to applications using non-proliferating cells. Along with safety, the main advantage of this approach is the availability of protocols for clinical translation of mRNA strategies, thanks in part to SARS-CoV-2 vaccine research. As of March 30, 2022, 64,4% of the worldwide population (49, 50), including the authors of this paper, have received at least one dose of Covid-19 vaccine and most of them thanks to advancements in nucleic acid delivery protocols. The lack of integration avoids the risk of genotoxicity associated with integrating vectors and transient expression safeguards against long-term toxicities, making this strategy a good approach to test the safety of first-in-human CARs, targeting molecules with expression in healthy tissues. The drawback of using transient gene transfer is the short-term potency that is counterproductive in strategies such as CAR T cell immunotherapy whose benefits are mainly associated with rapid *in vivo* expansion and generation of T cell memory and immunosurveillance. Conversely, mRNA delivery is a versatile, flexible, and safe means for all technologies involving a hit-and-run mechanism that requires only transient expression, such as for nuclease complex in gene editing, epitopes in vaccination, and transposase in stable nonviral gene transfer.

## 2 Purpose of the Review: Non-Viral Approaches for T Cell Engineering

Despite great enthusiasm followed the approval by FDA of CAR T cell products on the market, confounding challenges persist for products based on *ex vivo* lentiviral or gammaretroviral transduction. Since their introduction, transposon-based platforms seem to represent a feasible, cheaper and useful alternative to mediate gene transfer, and pioneer clinical studies are currently emerging. We aim here to explore the world of non-viral vectors navigating through their advantages and drawbacks. In the next paragraphs, we will be reviewing preclinical and clinical applications of the SB and PB transposons, the utilization of the mRNA, and the modalities to deliver non-viral vector into the cellular nucleus, such as electroporation and nanocarriers. We will then be discussing the critical aspects related to the safety and efficacy, with the intention to provide practical considerations for exploiting these tools in future clinical studies. Finally, we provide our vision for future gene therapy with the advent of novel challenges, such as multi-targeting design, but also innovative tools, including DNA nanovectors and improved gene-editing technologies. From this perspective, technologies such as CRISPR/Cas9 are expanding the possibilities available in the field of adoptive T cell therapy as reviewed in (51). Their application in combination with viral techniques falls beyond the scope of this review, whereas we will discuss the virus-free CRISPR-Cas9 approach in the session related to future directions. We are encouraged by the prospect of non-viral vectors simplifying the CAR T supply chain, making it less expensive, safer, and efficacious.

## 3 Sleeping Beauty

### 3.1 Vector Design and Delivery

Awakened after a long evolutionary “sleep”, SB was reconstructed from inactive transposon sequences present in fish genomes, becoming the first transposon to show activity in vertebrate cells (52), thereby leading new horizons for gene therapy [reviewed in (53–61)]. Based on classical Tc1/mariner DNA Class II TE, these “jumping” units are able to translocate from one genomic position to another through a cut-and-paste mechanism (62). The SB vector is constituted by two functional components: the transposon DNA, which carries the gene of interest flanked by ITRs, and the SB transposase, which recognizes the ITR sequences and mobilizes the transgene from the donor DNA to an acceptor site inside the genome (63, 64).

During the years, many attempts have been made to improve the design of the SB vectors, leading to the generation of several variants. Regarding the original transposon vector, referred to as pT, the modification of nucleotide residues within the ITR sequences by means of mutations, additions or deletions have give rise to in improved versions such as pT2, pT3, pT2B, and, lastly, pT4 (65, 66), which has an optimized donor vector architecture. Similarly, transposase has also been extensively optimized to increase the transposition efficiency. The first SB10 transposase has passed through different mutagenized hyperactive versions including the second-generation SB11 transposase, approximately threefold more active than the first-generation SB transposase, to the more recent SB100X, holding 100-fold increase activity than the first-generation enzyme (67). The hyperactive SB100X system has shown to allow for efficient and stable gene transfer in various cell types, including primary human T cells (68), in a non-homologous recombination restriction manner. Identification of the crystal structure of the transposase catalytic domain has recently allowed the design of hyperactive transposase variants, including the SB transposase mutant (I212S), named hySB100X, which has 30% higher transposition activity than SB100X (69). The enzyme can also be modified to catalyze the excision but not the genomic re-introduction, leading to extrachromosomal circles similar to the excision circles formed during the process of VDJ recombination. The exc+/int- mutant can be exploited for transient transgenesis, e.g. to remove reprogramming factors after generation of pluripotent stem cells (69). Similar to what have been implemented for the CRISPR/Cas9 systems, attempts have been made to deliver the SB100X as a protein. However, the SB transposases showed intrinsic protein instability, associated with low solubility as well as aggregating properties. For this reason, efforts to improve their chemical properties have led to the generation of a new highly soluble variant (hsSB), including the C176S and I212S substitutions, which has shown high self-penetrating properties (70). The efficiency of this new type of SB transposase was tested in human and mammalian cells such as stem cells, both of embryonic and hematopoietic origin, induced pluripotent stem cells (iPSCs), and primary cells such as human T cells. hsSB was able to generate anti-CD19 CAR T cells, even though with a lower transduction efficiency, displaying antitumor activity analogous to CAR T cells engineered with viral vectors in xenograft mice (71).

**Figure 2:**
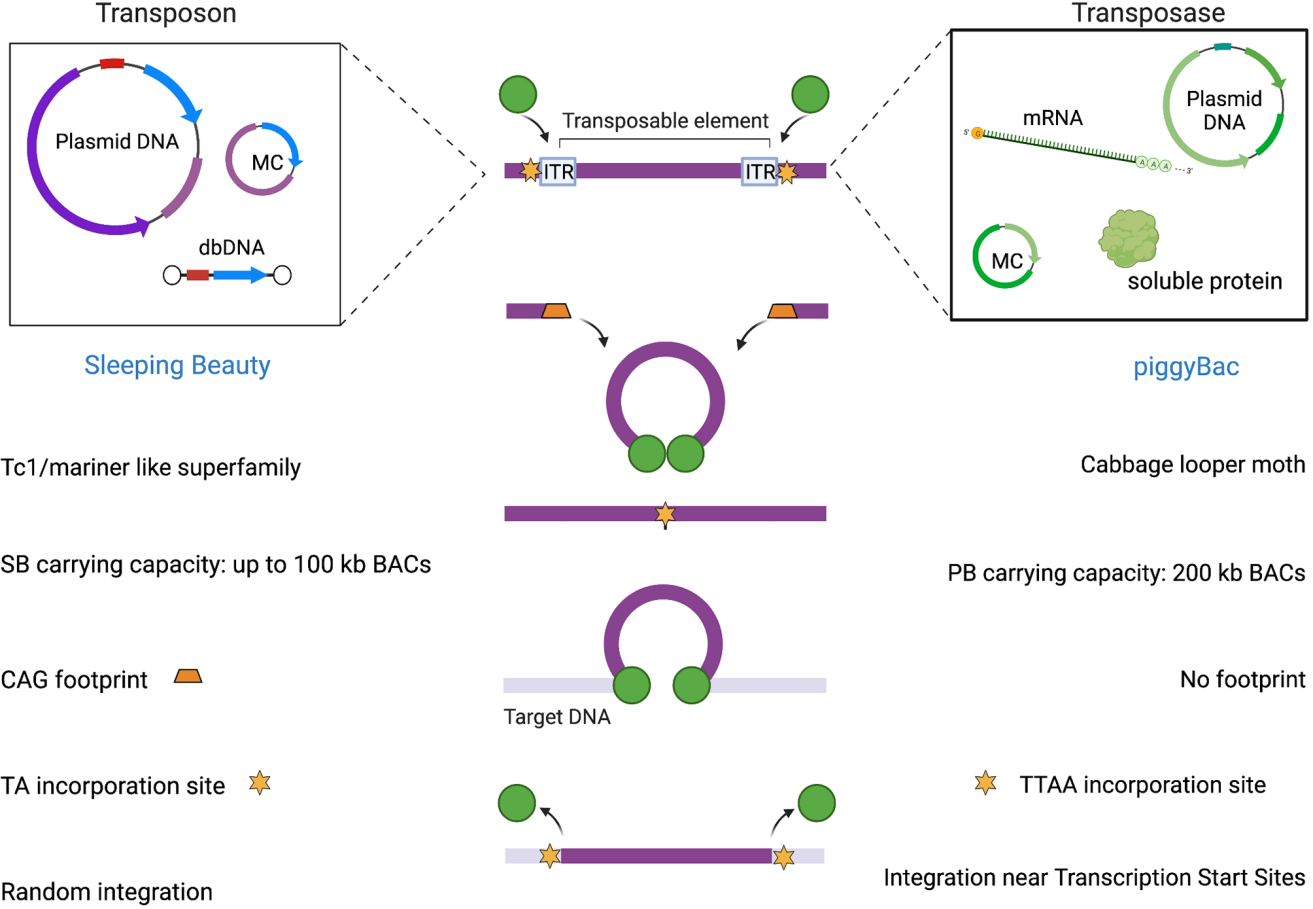
SB and PB mediated integration.

The integration of the excised transposon takes place in a close-to-random manner inside the genome when the transposase finds a target site characterized by a TA dinucleotide (72) as illustrated in **Figure 2**. When the transposase recognizes the ITR sequences flanking the SB donor transposon and binds them, it induces double-stranded breaks through the formation of a synaptic complex. The resulting excision site is rapidly repaired by host non-homologous end joining (NHEJ) and the terminal sequences of the SB transposon that are formed after the cleavage generate a characteristic footprint in the donor DNA. At this point, the transposon-transposase complex is free to find an appropriate target site in the genome and integrate inside, leading to target site duplication flanking the integrated element (73).

The peculiar integration bias has been deeply investigated through genome-wide integration analyses comparing SB with PB and Tol2 (74). The target site selection of SB, PB, MLV-derived gammaretroviral and HIV-derived lentiviral systems was compared in primary human CD4+ T cells. SB transposons demonstrated to have the highest probability to target safe harbors thanks to its unbiased, close-to-random integration profile as compared to other methods which instead showed a bias for transcriptional start sites, CpG islands and DNaseI hypersensitive sites (45). Therefore, the mechanistic features of SB make it a vehicle with a favorable risk-benefit assessment (45).

A big advantage of this type of strategy compared to viral systems is the greater cargo capacity, though there is an inverse correlation between the size of the insert and the efficiency of the transposition mechanism (75, 76). Optimal cargo size is under 6 kb but the sandwich version, comprising two complete transposon units flanking the cargo in an inverted orientation, favors an increase in load up to 11 kb thereby extending cloning capability of the SB based vectors (77, 78). Moreover, when combined with bacterial artificial chromosome (BACs), SB was shown to deliver transgenes up to 100 kb at reasonable efficiencies in human embryonic stem (ES) cells (79, 80).

Many attempts have been made to manage toxicity caused by the electroporation process. Since the damage is dependent on the amount of DNA delivered and the magnitude of voltage pulses in the electroporation process, the toxicity can be relieved by reducing the size of the SB vector and delivering the transposase in other forms than DNA plasmid. In this context, recent applications foresee the use of the SB transposase in the form of mRNA or recombinant protein and a minicircle vector (MC) encoding the transposon (71, 81). The transposase mRNA results in increased biosafety due to the fact that mRNA does not run the risk of chromosomal integration and allows transient expression of the enzyme. This aspect will be dealt with in more detail in paragraph 5.2. MCs are produced from plasmids through site-specific recombination to eliminate bacterial origin of replication and antibiotic resistance genes and retain exclusively the transgene with its promoter. The presence of antibiotic resistance gene in plasmids as a selection marker represent a safety concern for the risk of horizontal dissemination into pathogenic bacteria. MCs have a better transposition rate with a gene transfer 5-fold higher compared to conventional plasmids and additionally a lower toxicity (82). The use of bacteria plasmids can trigger an immune response by the host caused by the activation *via* Toll-like receptors by the unmethylated CpG motif in the bacterial DNA sequence. The lack of bacterial sequences inside MCs favors their lower immunogenicity (83). Unfortunately, there are currently no commercial large-scale GMP producers of MCs, hindering commercialization.

### 3.2 Preclinical Evidence

The ability of SB vectors to provide long-term expression *in vivo* has been demonstrated in multiple preclinical studies spanning a wide range of fields, ranging from the application in cancer to multiple diseases, reviewed by Hodge and colleagues (84).

The first representative studies that demonstrated the feasibility of SB technology to generate anti-CD19 CAR T cells from peripheral blood or cord blood were reported by Dr. Huang at the University of Minnesota, and Dr. Cooper at the MD Anderson Cancer Center (MDACC, Houston, TX, USA) (85, 86). They showed that SB transposase can be delivered either as plasmid DNA or mRNA in combination with a CAR-encoding transposon plasmid into T cells by electroporation to produce functional anti-CD19 CAR T cells. To achieve a high transduction of the final cell product, electroporated T cells were expanded by multiple stimulations with CD19+ artificial antigen-presenting cells (APCs), resulting in rapid outgrowth of CAR expressing T cells.

Our group developed a clinical-grade protocol to engineered T cells differentiated towards memory T cells with a CD8+CD56+ phenotype *in vitro*, namely cytokine-induced killer (CIK) cells with different CAR molecules (87), including the anti-CD19 CAR, anti-CD123 CAR, anti-BAFFR CAR, and anti-CD33 CAR. We transfected CIK cells with the SB11 transposase and the pT vector (44, 87). The choice of the T cell population was based on the high safety profile with minimal occurrence of graft-versus-host disease (GvHD) (88), that allows the use of donor-derived cells in clinical trials (89–91). In order to mitigate cell damage induced by electroporation, we developed an improved platform for SB-mediated engineering by stimulating electroporated T cells with irradiated autologous PBMCs as feeder cells. A single stimulation step allowed us to achieve a sufficient number of CAR T cells for clinical applications and up to 80% transgene expression in CIK cells as well as conventional T cells (44, 87). The key benefit of our methods is the limited manipulation, avoiding multiple stimulations. We confirmed the close-to-random distribution of integrations in engineered CAR T cells and the absence of integration near cancer related genes (87). Adoptive transfer of anti-CD19 CAR or anti-CD123 CAR lymphocytes led to a significant anti-tumor response in B-ALL and acute myeloid leukemia (AML) disseminated disease models, respectively. The preclinical evaluation phase demonstrated the possibility of generating with this platform CAR T cells characterized by a dose-dependent therapeutic effect in patient-derived xenograft models, in the absence of toxicity, through a robust and reproducible production process (44). Recently, the platform was applied to generate anti-CD33 CAR T cells by using the hyperactive SB100X transposase and the pT4 vector, which showed improved transduction efficiency compared to SB11 and pT systems and *in vivo* activity toward chemotherapy resistant/residual AML cells (92).

Another example of enhanced SB-mediated engineering is the one proposed by Monjezi et al. using SB100X and a pT2-based MCs. The author stimulated T cells with anti-CD3/CD28 beads before electroporation and the resulting transduction efficiency was about 30%. Prior to functional testing, EGFRt-positive CAR T cells were purified and expanded with irradiated CD19+ feeder cells. The resulting anti-CD19 CAR T cells have potent anti-tumor responses and was shown to be equally functional as anti-CD19 CAR T cells prepared by lentiviral transduction *in vitro* and *in vivo* (81).

In recent years, more and more functional advancements are taking place to reduce *ex vivo* manipulation of CAR T cells in order to preserve their persistence and anti-tumor activity (93). One of these efforts is represented by the study of Chicaybam and collaborators, demonstrating the possibility of generating SB-engineered anti-CD19 CAR T cells in 8 days activating with Transact (Miltenyi Biotec) after electroporation. The resulting cell populations exhibit robust antileukemic activity both *in vitro* and *in vivo* associated with a central memory phenotype (94). The same group demonstrated that CAR T cells can be generated by SB and used without the need of stimulation and expansion. Similar *in vivo* activity was demonstrated by CAR T cells injected 24 hours after electroporation and cells expanded with anti-CD3/CD28 coated beads for 8 days (95). This point-of-care technology can even be optimized by co-expression of a safety switch and a membrane-bound version of interleukin-15 (mbIL15) to enhance safety and *in vivo* persistence and demonstrated anti-tumor activity against CD19+ tumors and prolonged T cell survival in mouse models (96).

This approach has been utilized for the development of UltraCAR T platform based on the use of the non-viral system to deliver multiple genes by SB vectors. Using this platform, Chan et al., developed autologous cells co-expressing a CD33 CAR and mbIL15 (PRGN-3006) for the treatment of r/r AML and high-risk myelodysplastic syndrome (MDS). This platform shortened the manufacturing process and allows infusion of the product the day after transduction, obviating the need for *ex vivo* cells expansion. In preclinical validation, *in vivo* administration of a single dose of PRGN-3006 UltraCAR T cells significantly improved the overall survival of AML-bearing mice compared to CAR T cells lacking mbIL15 (97). The same group developed a PRGN-3007 UltraCAR T co-expressing mbIL15, a CAR specific for receptor tyrosine kinase-like orphan receptor 1 (ROR1) that is frequently overexpressed in hematological and solid tumors, a safety switch, and a novel mechanism for intrinsic blockade of PD-1 gene expression. Notably, preclinical data demonstrated the safety and the improved anti-tumor activity of PRGN-3007 compared with the control ROR1 CAR T (98).

The optimized donor-vector architecture of the pT4 vector coupled to the use of the hyperactive SB100X allows the generation CAR T cells engineered with bicistronic vectors. Using this platform, CAR T cells were incorporated with the inducible Caspase 9 (iC9) safety switch and showed anti-leukemic activity in mouse models and were as efficient as CAR T cells generated with a LV vector (99). Furthermore, this system was used to combine the expression of anti-CD33 CAR and the chemokine receptor CXCR4 to increase CARCIK cell homing to the bone marrow niche (100).

Recent evidence reveals the suitability of the SB vector to enable engineering of primary natural killer (NK) cells with anti-CD19 CAR, which showed a safe genomic integration profile and antitumor activity *in vivo* (101). Manufacturing protocols associated with preclinical studies employing SB in the context of CAR T cells are summarized in **Table 2**.

**Table 2:** Manufacturing protocols associated with preclinical studies employing SB in the context of CAR T cells.

Background	Description	Vector	Electroporation	Stimulation	Transduction and Yield	Reference
B-ALL and AML	Anti-CD19 CAR-T cells showed proof-of-concept tumor eradication in B-ALL xenograft models; anti-CD123 CAR T cells controlled KG-1 AML in xenograft models	CD19CAR/pT MNDU3 transposon (15 μg) + pCMV‐SB11 transposase (5 μg)	10^7 peripheral blood mononuclear cells (PBMCs) using 4D‐Nucleofector system (Program EO-115) with P3 Primary Cell 4D‐Nucleofector X kit (Lonza)	Autologous PBMCs irradiated with 60Gy γ‐rays are added after electroporation and OKT‐3 is added at day 1. IL‐2 is added weekly.	CAR expression: 75.6% for CD123.CAR and 80% for CD19.CAR Large scale T cell expansion: 23-fold in 18-21 days CAR T cell expansion: from 60×10^6 to 8.6×10^8	Magnani et al; 2018 (44)
AML	Anti-CD33 CAR-T cells showed delaying AML progression in patient-derived xenograft models	CD33CAR/pT transposon (15 μg) + SB100X-pT4 transposase (0.5 μg)	As Magnani et al.;2016 (87)	As Magnani et al.;2016 (87)	CD33CAR expression: 63.7%T cell expansion: 38.8-fold after 3 weeks.	Rotiroti et al.;2020 (92)
Glioma	Production of EGFRvIII CART cells in two weeks showed superior therapeutic efficacy in mice bearing established orthotopic gliomas	EGFRvIIICAR pT/neo transposon (10 μg) + pCMV-SB11 transposase (5 μg)	20 × 10^6 PBMCs or T cells using Amaxa Nucleofector 2B (Lonza) with program U-014 in T-cell electroporation buffer (Lonza)	CAR T cells were stimulated with 100 Gy-irradiated EGFRvIII+ K562 ells in the presence of IL-21. After7 days, T cells were restimulated in the presence of IL-2 and IL-21.	CAR T cell (back calculated inferred numerical expansion): From 10^6 to around 5×10^7 in two weeks and 10^9 in 30 daysEGFRvIII CAR expression: around 90%	Caruso et al.;2019 (102)
CD19+ B-cell malignancies	Anti-CD19 CAR-T cells generated in 8 days showed effective antitumor response in mice xenografted with RS4;11 or Nalm-6 B-cell leukemias	pT3 19BBz CAR transposon (20 μg) + pCMV-SB100x transposase (1 μg)	10^7 PBMC using Amaxa Nucleofector 2B with program U-14	After electroporation cells are cultured with IL-2 and, 2h later, are activated with T Cell TransAct (Miltenyi Biotec)	Absolute number of T‐cell expansion: from 10^7 to 3.6×10^7 after 8 days of cultureCAR expression range: 20.4%–37.3%	Chicaybam et al.;2020 (94)
Lymphoma	anti-CD19 CAR T cells engineered with MC SB vectors eradicated lymphoma cells in Raji xenograft model	pT2 CAR EGFRt MC DNA (1 μg) +pCMV-SB100X MC (1:1 ratio) or pCMV-SB100X mRNA (1:4 ratio)	T cells are activated with anti-CD3/CD28 beads (Thermo Fisher Scientific) and on day 2 electroporated (1×10^6 T cells) using 4D-Nucleofector	After electroporation T cells are propagated with IL-2. Prior to functional testing, EGFRt-positive T cells are enriched and expanded with irradiated CD19+ feeder cells for at least 7 days	CAR T cell expansion: From 1×10^6 to around 20×10^6 (MC-MC) and 12×10^6 (MC-mRNA) in 2 weeksCAR expression: 49.8% for MC-MC and about 40% for MC-mRNA on day14	Monetzi et al;2017 (81)

### 3.3 Clinical Applications

Following promising results obtained in the preclinical phase, the group of Cooper et al. contributed to the clinical debut of SB-engineered anti-CD19 CAR T cells and provided proof of concept of the convenience of SB transposition for CAR T cell engineering. Two pilot clinical trials (NCT00968760, NCT01497184) confirm the safety of SB-engineered anti-CD19 CAR T cells in 26 patients with B-ALL and non-Hodgkin’s Lymphoma as adjuvant therapy after autologous or allogeneic hematopoietic stem cells transplant (HSCT) (103). Cell product manufacturing included T cell nucleofection with the transposase SB11 plasmid and pT2 vector encoding a second generation anti-CD19 CAR with CD28 as a costimulatory agent and *ex vivo* propagation for approximately 28 days with multiple stimulations using artificial APCs and cytokines. Patients were subsequently enrolled in a long-term follow-up study lasting up to 15 years and the persistence of genetically modified T cells was monitored annually using droplet digital polymerase chain reaction (ddPCR) and flow cytometry. Limited expansion and absence of B-cell aplasia were reported. However, CAR+ T cells were detected up to 4 years after infusion in autologous HSCT recipients and 2 years in allogeneic HSCT recipients (104). Since long manufacturing processes and multiple stimulations are known to impair T cell fitness, resulting in decreased efficacy *in vivo*, in collaboration with Ziopharm Oncology (Boston, MA), a second-generation approach was developed by reducing to 2 weeks the time require for coculture with feeder cells. A clinical trial was designed (NCT02529813) in which CAR T cells have been infused in combination with a Fludarabine- and Cyclophosphamide-based lymphodepletion regimen in adult and pediatric patients with active CD19+ malignancies (105). This trial aim is to provide data supporting a 3rd-generation point-of-care trial to very rapidly manufacture (< 2 days) anti-CD19 CAR T cells in absence of feeder cells (96).

Thanks to the in-house establishment of a clinical-grade platform to obtain non-viral CAR T cells in about 20 days (44), we designed a multicenter phase I/II trial in B-ALL patients relapsed after allogeneic HSCT (NCT03389035). Donor-derived anti-CD19 CAR T cells were generated by electroporation with the SB11 transposase-encoding plasmid and a transposon expressing a third-generation CAR and differentiation into CIK cells (CARCIK-CD19). Cells were manufactured from 50 mL of peripheral blood from the allo-transplant donor. A total of 21 patients, 4 children and 17 adults were lymphodepleted and treated with a single infusion of CARCIK-CD19 product. In most patients, potent CAR T cell expansion and long-term persistence were achieved, which was associated with anti-leukemic activity and induction of a sustained response. Moreover, integration site analysis performed on patients’ peripheral blood demonstrated that SB integration pattern, with absence of preference for transcriptional start sites and promoters, is maintained after infusion. High polyclonal marking and population diversity confirmed the positive safety profile of the SB technology (43). Cytokine release syndrome (CRS) was observed in six patients and neurotoxicity in two patients while acute GvHD was never observed (106). As a reinforcement of the previously implemented study, a new trial in our centers that involves re-treatment of patients has recently begun and patient enrollment is currently underway.

Besides the reported trials, CAR T studies using the SB platform are currently underway in the USA and Europe.

Given the promising preclinical data of UltraCAR T cells, two clinical trials have been launched. Specifically, a Phase 1/1b first-in-human dose escalation/dose expansion study (NCT03927261) is evaluating the safety of PRGN-3006 UltraCAR T co-expressing an anti-CD33 CAR and mbIL15 in adult patients with r/r AML, hypomethylating agents (HMA) failure, high risk MDS and chronic myelomonocytic leukemia (CMML). Preliminary data showed that PRGN-3006 infusion was well tolerated and achieved a 50% response rate in patients treated with lymphodepletion, associated with CAR T cell expansion and persistence (107).

A second study (NCT03907527), evaluating the safety of PRGN-3005 UltraCAR T cells co-expressing an anti-MUC16 CAR, mbIL15 and a kill switch in the treatment of patients with platinum resistant ovarian cancer patients is ongoing (108).

The CARAMBA trial has been recently launched as a joint effort supported by an EU Horizon grant and is using mRNA encoding the hyperactive SB100X transposases in conjunction with CAR transposon supplied as an MC vector. In this Phase I/II clinical trial autologous anti-slam family member 7 (SLAMF7) CAR T cells are being used against MM to investigate the feasibility, safety, and anti-myeloma efficacy (109).

## 4 PiggyBac

### 4.1 Vector Design

PB was originally isolated from the cabbage looper moth *Trichoplusia ni* over 30 years ago and has been optimized over the years (110). As with the SB vector, the PB system is constituted by the PB transposase (PBase), in the form of mRNA or DNA, and a separate transfer plasmid carrying the desired genetic cargo (111). It belongs to the class of DNA transposases and to improve its transposition efficiency, the transposase has been optimized through random mutations resulting in useful variants such as the hyperactive version of PBase (hyPBase) (112). Another interesting variant is the excision competent/integration defective (exc+int-) PB transposase that allows transient transgenesis, by enabling excision in the absence of re-integration into the host genome. One potential application of exc+int- PBase would be the transient introduction of transcription factors for transgene-free iPSC production, the same as for the exc+/int- mutant of SB100X. The exc+int- PBase can be fused to zinc finger proteins binding to safe harbors to favor integration into to specific genomic regions (113).

The design of PB transposon vectors is characterized by a single open-reading frame (ORF) flanked by ITRs that in PB are characteristically asymmetric. The transposase recognizes ITRs flanking the transposon and catalyzes transgene excision and integration into genomic DNA by a cut-and-paste mechanism. Specifically, the transposition involves a series of hydrolysis and transesterification reactions with the generation of a DNA intermediate in which DNA hairpins provide exonuclease protection for the transposon ends. One of the peculiar features of PB is its specificity towards TTAA sites for integration in contrast to SB’s preference for TA dinucleotides. Although PB can integrate into any TTAA target site, the epigenetic status may affect integration site preference of PB transposons. Another attractive characteristic of the PB transposase is the lack of a DNA footprint after its excision (see **Figure 2**). In contrast to the mobilization of other conventional DNA transposons like SB, which are associated with NHEJ of the donor DNA (114), PB does not require DNA synthesis. Indeed, as long as an active transposase persists in the cell, integrated transposons can be remobilized to new sites. In the event that the transposon has integrated into a gene, the footprints created at the excision site could produce undesirable mutations of the gene in which they were left. This feature is an advantage of the PB system from the safety perspective.

PB has a higher transposition activity for transposon mobilization than SB in mammalian cells (115), a larger cargo capacity (up to 14 kb) than viral vectors, and allows multiple transgene delivery through the design of multicistronic cassettes (116). Moreover, like gammaretrovirus, PB showed a preference for integration near TSSs, CpG islands and DNaseI hypersensitive sites, the consequence being that the risk of gene dysregulation is increased (73). Furthermore, analyzing integration sites occupied under the selective pressure provided in insertional mutagenesis (IM) screens, it has been demonstrated that PB compared to SB, is more prone to association with oncogenes (117).

Recently, the discovery in human genome of the human piggyBac transposable element derived 5 (PGBD5) has raised possible safety concerns in PB-gene transfer application (46, 118). Indeed, the presence of PGBD5 could allow the remobilization of PB transposons in human cells with a higher risk of genes dysregulation. With respect to this aspect, however, there is still considerable uncertainty. Beckermann T. M. et al., observed that transposition activity is probably restricted within species to cognate ITR sequences and in particular, PGBD5 appeared in their study, unable to bind, excise or integrate PB transposon in human cells (119).

Therefore, PB has a series of useful characteristics for genetic engineering: i. a higher transposition activity than SB, ii. its precise excision from an insertion site, restoring the site to its pre-transposon state without DNA footprint, iii. its wide range of applications such as mutagenesis, introduction of reprogramming factors to generate iPSCs, and gene transfer. For the purpose of the review, we focus on the application of PB transposon system in the CAR T production.

### 4.2 Preclinical Evidence

One of the most challenging issues using transposons is the toxicity of the transduction procedure. In particular, electroporation in the presence of exogenous DNA is toxic and decreases cell survival to less than 40% after 24h from transfection. To improve the efficiency of PB transfection, different approaches have been tested such as the addition of survival-promoting cytokines such as IL-7 or IL-15 that increase the frequency of gene expression and the ability of the transduced cells to expand. Alternatively, T cell expansion was stimulated by the use of feeder cells represented by autologous PBMCs or other sources such as the K562 cells, modified to express costimulatory molecules. Therefore, the quality of the final CAR T product depends on several factors that go from the construct characteristics (such as cargo size, costimulatory domains, spacers) to the manufacturing platform. Many preclinical studies exploited PB as a tool to generate CAR T cells for hematological malignancies and solid tumors.

One of the first pieces of evidence of the potential for the PB platform to stably transfect human T cells in cancer therapy were reported by Nakazawa Y. et al. They obtained stable gene expression in about 20% of primary T cells without selection, improved to 40% with the addition of IL-15 (120). In a subsequent study by the same group, a significant increase in CAR expression was achieved using irradiated activated T cells as feeders and alternative means of TCR stimulation using viral antigens instead of anti-CD3/CD28 mAbs. Efficiency was further improved by reducing the size of the CAR cassette with the elimination of the long IgG1.CH2CH3 spacer (121). With this approach, PB-generated anti-CD19 CAR T were used to treat B-ALL cells in the central nervous system (CNS) in a xenograft mouse model comparing intra-venous and intra-cerebroventricular delivery. Direct CNS delivery of CAR T cells resulted in eradication of B-ALL from the CNS without fatal adverse events, proving the activity of PB-generated CAR T cell *in vivo* and suggesting this strategy as a possible therapeutic approach for isolated or advanced CNS disease (122).

The use of feeders to support generation of CAR T electroporated with non-viral transposons appears to be useful also for PB. Similar to what we first described for generating CAR T cells with SB (123), irradiated autologous PBMCs have been used to efficiently produce CAR T cells (124). Although the generation of anti-CD19 CAR T cells with the PB transposon system was demonstrated to be efficient using feeders, first attempts showed poor *in vivo* activity due to the interactions between the CAR spacer and Fc gamma receptor-expressing cells. Optimization of the construct led to the generation of an anti-CD19 CAR lacking the spacer IgG1 Fc region which demonstrated superior efficacy in a murine B-ALL xenograft model. Moreover, the inclusion of 4-1BB costimulatory domain had greater efficacy *in vitro* and *in vivo* at lower CAR T cell doses than those with a CD28 costimulatory domain (111).

Most of the manufacturing protocols for viral CAR T production activate T cells with anti-CD3/CD28 stimulation and the addition of IL-2 during culture. This system may have some limitations when applied to cells electroporated with transposons, such as the expansion of non-transduced T cells and the enrichment of terminal effector T cells at the expense of the immature stages. An alternative approach is the activation of the CAR T receptor by its cognate ligand or specific anti-CAR antibody in the presence of IL-4 and IL-7, which led to selected expansion of functional anti-CD19 CAR T cells, resulting in 90% of CAR positive cells. Moreover, the addition of IL-21 to the IL-4 and IL-7 mixture improves the immunophenotype of CAR T cells with more represented immature stages with less expression of exhaustion molecules such as PD-1, LAG-3, and TIM-3 (125).

CAR-mediated stimulation is often required to obtain sufficient numbers of CAR+ cells. For instance, none of the previously reported methods, including HER2-expressing tumor cells, irradiated activated feeder T cells with anti-CD3/CD28 antibodies, and autologous irradiated PBMCs alone, was able to improve the expansion of anti-HER2 CAR T cells modified with PB. Conversely, stimulation with autologous PBMCs engineered with HER2 and costimulatory molecules such as CD80 and 4-1BBL enhanced the expansion of anti-HER2 CAR T cells modified with PB. At the end of the expansion, the cellular product was enriched in CAR T stem cell memory-like cells and exerts anti-leukemic activity *in vitro* and *in vivo* (126).

Another manufacturing platform developed to reduce T cell exhaustion applied PBMCs pulsed with a pool of viral peptides and IL-7 and IL-15 in the first week, followed by stimulation on anti-CD3 or anti-CD28 mAbs-coated plates. With this protocol, in the setting of neuroblastoma, functional anti-GD2 CAR T cells were associated to low expression of PD-1 and improved naïve/stem cell memory phenotype. In addition, the authors suggested a possible synergistic effect of PB anti-GD2 CAR T cells and MEK inhibitors (i.e. trametinib) regardless of the mutation status of the MAPK pathway in tumor cells with an enhanced efficacy of CAR T therapy in the setting of neuroblastoma (127).

CAR T cells directed against granulocyte-macrophage colony-stimulating factor receptor (hGMR or CD116) generated with PB were used in a non-human primate (NHP) model to evaluate their safety (128). To generate cynomolgus macaque CAR T cells, electroporated PBMCs were cultured in the presence of human IL-15 and IL-7 with the addition of immature dendritic cells, derived from autologous cynomolgus PBMCs using human IL-4 and GM-CSF.

The anti-hGMR CAR T design has been further optimized by substitution of the antigen-binding domain with a mutated GM-CSF and CH2CH3 hinge with a G4S spacer and an improved anti-tumor activity against CD116+ AML was demonstrated both *in vivo* and *in vitro* (129).

Another issue with classic transposon transduction protocols is their reliance on bacteria for the production of plasmid vectors. To avoid undesired qualities of bacterial plasmids, including activation of host immune responses, antibiotic resistance, and endotoxins, CAR+ T cells were produced by co-electroporation of a linear DNA transposon and mRNA encoding the PB transposase, reaching a transfection efficiency of 60% and a vector copy number (VCN) of less than 3 copies of transgene per transduced cell. The linear vector was prepared enzymatically *in vitro* by PCR whereas mRNA was obtained through *in vitro* transcription. Electroporated cells were cultivated in presence of IL-4, IL-7, and IL-21 and maintained an early memory immunophenotype at the end of the differentiation (125). Similarly, the possibility to include the gene of interest flanked by ITRs in doggybone DNA vectors (dbDNA) was investigated. dbDNA are synthetic, linear, covalently closed DNA vectors that can be inexpensively and rapidly produced *in vitro* at large scale in a bacteria-free system from the parent plasmid. Unlike open-ended linear DNA which had a propensity for integration, dbDNA with their covalently closed ends has a lower tendency to integrate with a reduced risk of undesirable genomic integration of PB transposase. Using two linear dbDNAs containing PB transposase and the anti-CD19 CAR cassette incorporating 200bp sequences flanking the ITRs, respectively, it was possible to produce CAR T cells *in vitro* (130). The manufacturing protocols for PB-generated CAR T cells are summarized in **Table 3**.

**Table 3:** Manufacturing protocols associated with preclinical studies employing PB in the context of CAR T cells.

Background	Description	Vectors	Electroporation	Stimulation	Transduction and yield	Reference
PB transposon platform	Optimization of PB transposon platform for T-cells engineering using GFP as reporter	different quantities of pIRII-eGFP and pCMV-PB transposase	5x10^6 PBMCs using Nucleofector Device (Lonza, program U-014) with the human T-cell Nucleofector Kit	stimulation with CD3/CD28 mAbs and cytokines (IL-2, IL-15, IL-7, IL-4); transgenic T cells were selected on day 8 and expanded with feeder cells (autologous PBMCs or modified K562 cells)	Optimal results were obtained with 5µg of transposon and transposase with a transfection efficiency of 20%, improved to 30-40% with addition of IL-15.	Nakazawa et al., 2009 (120)
B-ALL	anti-CD19 CAR-T cells lacking the spacer IgG1 Fc region demonstrated superior efficacy in murine B-ALL xenograft models	pVAX1PB (5µg) + pVAX1SPBase (5µg)	4x10^6 PBMCs using Neon Electroporator with single pulse, 20 ms and 2400 V	Electroporated cells were cultivated in presence of IL-15 and stimulated with irradiated autologous PBMCs on D1 and after every 7 days.	Expansion: 100-fold after 22 days CAR expression: from 35% to 97%, depending on the construct	Bishop D. C. et al., 2018 (111)
CD19+ B-cell malignancies	Anti-CD19 CAR T cells manufactured in the presence of IL-4, IL-7 and IL-21 showed effective cytotoxic activity *in vitro*	5 µg (2:1 mixture of PB transposon vector and pCMV-PB hyperactive-transposase)	4x10^6 PBMCs using Neon electroporator inbuffer T (1x20 ms/2300V)	Electroporated cells were stimulated the day after in the presence of IL-4, IL-7 and IL-21 (stimulation by CD19 expressed on the surface of B cells in PBMC)	CAR expression: 90% in the presence of IL-4, IL-7 and IL-21. 30% when stimulated with anti-CD3/CD28 mAbs Expansion: from 4x10^6 to about 30-40×10^6 in 17 days and 100-120×10^6 in 24 days	P. Ptackova et al., 2018 (131)
CD19+ B-cell malignancies	anti-CD19 CAR T cells generated with co-electroporation of linear DNA transposon and mRNA encoding transposase showed lytic activity *in vitro*	pPB DNA linear transposon produced by PCR (3-0,3µg) + hyPBase mRNA transposase (12 µg) with 3′-O-Me-m7G (5′) PPP(5′) G RNA cap structure	1x10^7 PBMCs electroporated as in Ptackova et al., 2018 (131)	stimulation with TransAct reagent the day after electroporation and expansion for 21 days in the presence of IL-4, IL-7, and IL-21	CAR expression: 60-70% after 14-21 day of expansion Expansion: from 1x10^7 to 1x10^7 in 14 days and 1x10^8 in 21 days	I. Kastankova et al., 2021 (125)
Neuroblastoma	Anti-GD2 CAR T cells manufactured using autologous PBMCs pulsed with a pool of viral peptides showed effective antitumor response in xenograft model when combined with MEK inhibitor	pIRII-GD2-28Z CAR plasmid (7.5µg) + pCMV-PB transposase plasmid (7. 5µg)	2x10^7 PBMCs using 4D-Nucleofector and the P3 Primary Cell 4D-Nucleofector X kit, program FI-115 [See Morita D. et al., 2018 (121)]	stimulation with 5x10^6 autologous PBMCs pulsed with MACS PepTivator (AdV5 Hexon, HCMVpp65, EBNA-1, and BZLF), IL-7 and IL-15. Transfer to anti-CD3 or anti-CD28 mAb-coated plates on day 7 and expansion in G-Rex 6 Multi-Well Cell Culture Plates (Wilson Wolf Corporation, New Brighton) on day 9	CAR expression: 44% ± 6% at day 14 after transfection.	Tomida A. et al., 2021 (127)
HER2 positive solid tumor	HER2-CAR-T cells showed the ability to control Her2-positive tumor in mice	pIRII-HER2-28z plasmid (5µg) + pCMV-PB transposase plasmid (7.5µg)	20x10^6 PBMCs using 4D-Nucleofector and the P3 Primary Cell 4D-Nucleofector X kit, program FI-115 or the MaxCyte ATX protocol RTC 14-3.	Electroporated cells were stimuled with PBMC, electroporated with plasmid encoding tHER2, CD80 and 4-1BBL and UV-inactivated, on day 1 and cultivated in presence of IL-7 and IL-15 for 14 days	Expansion: 8 ± 1 fold CAR CAR expression: 60% ± 9% at day 14.	Nakamura K. Et al, 2021 (126)

### 4.3 Clinical Applications

Growing preclinical data supporting the feasibility and safety of the PB-based platform for CAR T manufacturing have allowed this system to enter clinical trials. The Australian CARTELL trial is a phase-I study (ACTRN12617001579381) to investigate the efficacy and safety of donor-derived anti-CD19 CAR T cells obtained through the PB transposon system in patients with relapsed and refractory CD19+ B-cell malignancies after HLA-matched sibling HSCT. Early results suggested activity similar to that of anti-CD19 CAR T cells generated with viral vectors with a high response rate. However, two of 10 treated patients developed malignant CAR 19 T cell tumor, one of whom died of disease-related complications while the other patient was successfully treated (132). Malignant cells showed high transgene copy number (24 copies) in the first reported patient, CAR overexpression, alteration in genomic copy number variation, and insertion into the *BACH2* and *FYN* genes. It is not yet clear which event caused the CAR T cell transformation, but it is widely accepted that the probability of insertional oncogenesis increases as the transgene copy number increases, which is why a limit of 5 VCNs is normally required by the regulatory authorities. Furthermore, it is likely that the numerous genomic deletions and insertions observed may have been driven by the use of a single high voltage pulse or excessive transposase activity. Insertional mutagenesis may also have contributed to the process of transformation. Both patients who developed lymphoma have an intronic integration in the *BACH2* gene, whose expression is therefore downregulated. BACH2 is a DNA-binding and transcription-regulating protein that plays a key immunoregulatory role and has been previously associated with cutaneous T cells lymphomas (133). *BACH2* is one of the genes most frequently targeted by HIV-1 insertion but HIV integration into *BACH2* has never been associated with insertional mutagenesis (134). Hence for now there is no clear evidence of the contribution of these integrations to transformation.

Two phase I studies conducted respectively in Japan (UMIN Clinical Trials registry ID: UMIN000030984) and China (clinicaltrials.gov ID: NCT04289220) are investigating the feasibility and safety of anti-CD19 CAR T cells manufactured with the PB system. In the Japanese study, three patients with R/R B-ALL were infused with 1x10^5 autologous anti-CD19 CAR T cells per kilogram after lymphodepletion in cohort 1. All patients previously received HSCT. Interestingly, administration of T cells produced by PB was safe and none of the patients showed dose-limiting-toxicities so far. One patients showed a B-cell aplasia lasting 9 months (135).

Results from a phase I trial using PB-generated anti-EGFR CAR T in R/R advanced non-small cell lung carcinoma (NSCLC) were recently published. Nine patients were treated with anti-EGFR CAR T cells, without grade 4 adverse events. Despite most patients showing the presence of circulating CAR T cells, only one patient showed a partial response while the other patients had persistent disease or progressed (NCT03182816) (136).

Although the results of transposon-engineered CAR T cells in clinical trials are preliminary, several early signs of clinical efficacy are emerging. A step forward has been made with anti-BCMA CAR T cells (P-BCMA-101) engineered through the PB platform for patients with R/R MM. To improve transposition, the manufacturing process was changed during the study to include the use of nanoplasmids that allow for the reduction of backbone size and bring ITRs closer. The cellular product showed a high composition of T stem cell memory (TSCM). Ninety patients have been treated with P-BCMA-101 and early results showed an overall response rate (ORR) 57% in the initial dose escalation and 73% in combination with Rituximab with remarkably low toxicity (clinicaltrials.gov ID: NCT03288493) (137).

## 5 mRNA

RNA has emerged as a versatile therapeutic reagent (138, 139). Seminal work by Malone more than 30 years ago demonstrated that RNA mixed with lipids can be absorbed by human cells and translate protein from it (140). Malone postulated in 1988 that if cells can create proteins from external mRNA then it might be possible to “treat mRNA as a drug”. Since then, RNA has been used in other ways such as to restore functional expression of a mutated gene, knock out genes to silence expression (141, 142), modify cell phenotypes or to encode antigens. Here we focus on the use of RNA to modify leukocytes (143) to achieve temporary or long-term expression of CAR receptors in T cells.

### 5.1 Vector Design

Successful protein expression from RNA depends on its stability and translational efficiency. Those features are determined by cis-acting elements such as a 5’ cap structure, polyA tail, and the composition of the coding sequence as well as untranslated regions that might be present on 5’ and 3’ ends of the molecule. These cis-acting elements in the RNA conspire with trans-acting cellular factors leading to translation and protein production.

The sequence of the RNA cassette can be encoded by a linearized DNA plasmid or by a PCR fragment that contains an RNA polymerase binding site or promoter, such as the bacteriophage T7 RNA polymerase to initiate the transcription reaction. Tools are available for coding sequence optimization including codon optimization for changing synonymous codons for enhanced expression in target tissues or cells, reducing secondary structures of the RNA that lower translation levels (144) or modification of the coding sequence itself to express more active isoforms (145, 146). More recently, producing RNA molecules with modified ribonucleosides such as pseudouridine has demonstrated improved translational capacity as well as diminished immunogenicity by decreased stimulation of Toll-like receptors (TLRs). This makes it particularly useful for work with immune cells, such as T cells, that express TLRs or as a vaccine (147). The first-in-man data for the use of RNA with modified 1-methyl pseudouridine became widely available with the advancement of mRNA Covid-19 vaccines (148, 149).

The cap structure at the 5’ end of the RNA molecule, required for translation, can be incorporated in a co-transcriptional manner, using the m7G(5’)pppG cap analog (150), Anti-Reverser Cap Analog (ARCA) (151), by enzymatic methods such as recombinant vaccinia virus capping enzyme (152, 153) or by a more advanced technology called CleanCap^®^ which results in a natural structure of the RNA cap (type 1 cap).

A PolyA tail can be encoded in a transcription template containing a stretch of 64T nucleotides at the 3’ end of the molecule (146). Some RNA molecules (145) require longer polyA tails for efficient expression and biological activity. However, the longer polyA tail cannot be encoded in the plasmid template due to the instability of long homopolymeric stretches in plasmid DNA (154). A longer polyA tail can be added using enzymatic polyadenylation. We and others reported that a longer poly(A) tail of 120 A residues as opposed to the more conventional poly(A) tail of 64 bases achieves higher protein expression levels (145, 155).

**Figure 3:**
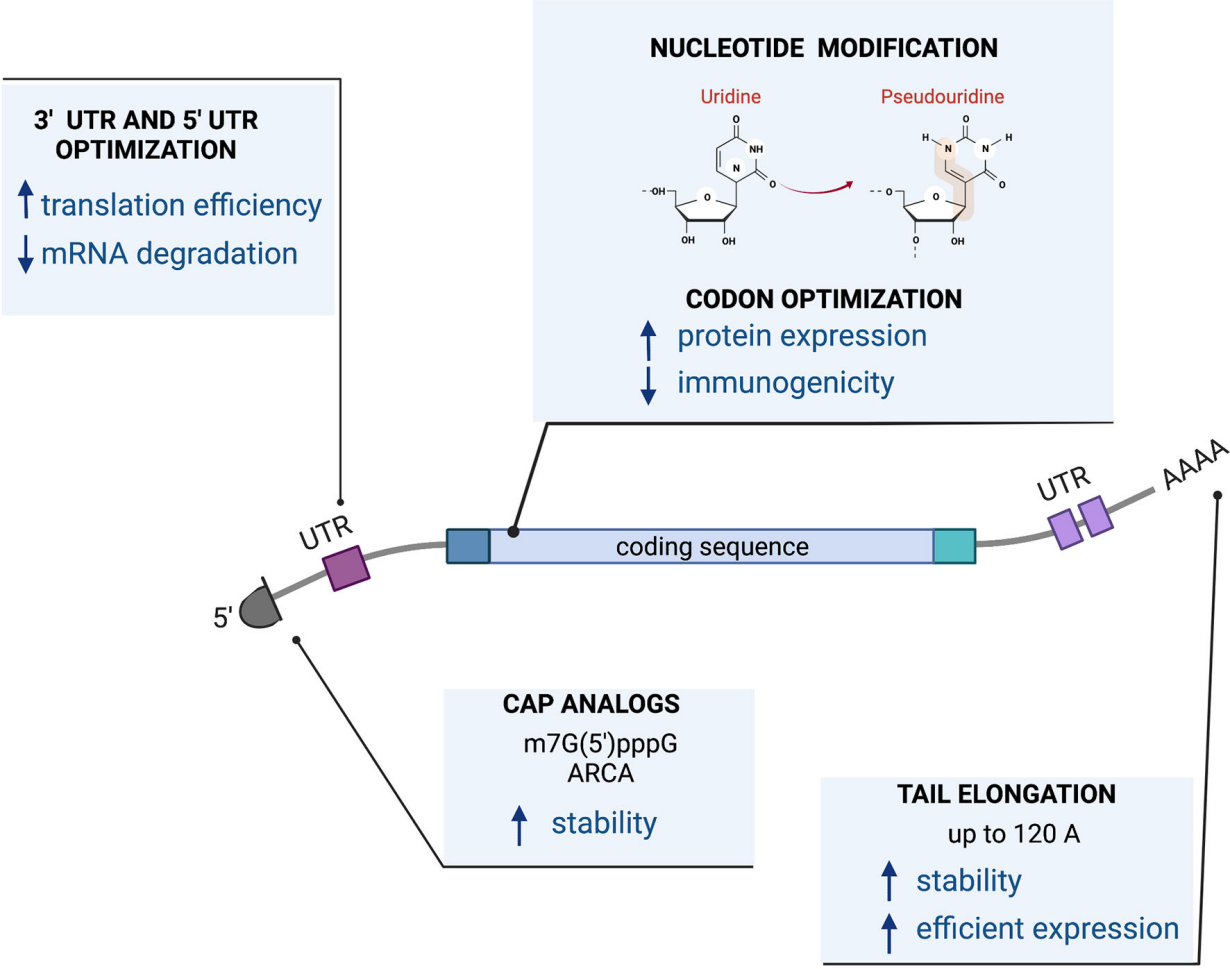
mRNA vector.

Additionally, both the 5’ and 3’ ends of an RNA molecule can be further modified with flanking untranslated regions (UTRs) to enhance translation (155–161). **Figure 3** presents the design of a mRNA vector.

### 5.2 Preclinical Evidence

RNA lends itself to versatile transfection methods with cells including electroporation (162), cationic lipids (163), and cationic polymers (164). mRNA has been used in a number of *in vitro* and *in vivo* preclinical studies to introduce CARs into T cells for testing in model systems for hematological tumors chronic lymphocytic leukemia (CLL), AML, ALL and solid tumors. Cytotoxicity and tumor growth inhibition was demonstrated in these models (165, 166). While mRNA-based therapies were shown to have reduced off target effects, lower toxicity and alleviate integration-associated safety concerns, the transient nature of protein expression was also a disadvantage in these applications. CAR constructs introduced into T cells with RNA was shown *in vitro* to last for 7 days (167) in absence of proliferation, and would limit the ability for the functionality of modified cells to persist.

A different approach to genetic modification of lymphocytes is to deliver a transgene of interest in the form of DNA together with RNA encoding a transposase enzyme. The first reported demonstration of successful gene transfer using an mRNA-encoded transposase was in the SB11 system (168). It was shown that SB11 transposase RNA stabilized with 5’ and 3’ untranslated sequences of the *Xenopus laevis* beta-globin gene successfully integrated a puromycin resistance gene from a pT2/PGK-Puro DNA plasmid in HT1080 human epithelial cells *in vitro*. The transposition efficiency as measured by puromycin resistance was greater using SB11 encoded by DNA compared RNA. The number of puromycin-resistant colonies per 10^6 cells plated was greatest with SB11 DNA under a UbC promotor (40X increase over a no SB11 control) followed by SB11 DNA under a PGK promotor (23X) and SB11 RNA using the PGK promotor (9X).

There are several advantages to encoding transposase enzymes in the form of mRNA when co-transduced into target cells along with a DNA vector encoding the gene of interest (168–170). One of the benefits of mRNA-based expression of a transposase is that its narrow window of transposase expression reduces the rate of secondary transposition events, which are caused by re-excision and re-integration of the transposon (171). Work conducted with a hyperactive form of Sleeping beauty, SB100X RNA, generated evidence that transposase can be used to efficiently integrate CARs into genomes of human T cells. An advantage of this approach is that the ratio of SB mRNA and DNA CAR construct can be precisely titrated to achieve durable integration with a low number of integrations per genome (68). The use of mRNA was also shown to allow for a transient, dose-controlled expression of SB100X in the absence of cytotoxic effects in various cell types (172). Another form of transposase, PB delivered in the form of mRNA was also shown to genetically modify HeLa cells when co-transfected along with a DNA plasmid encoding the neomycin resistance gene (170). Similar to the outcome in the SB system, using an RNA-encoded transposase, the PB transposase RNA was less efficient by a significant margin at transposition compared to PBase encoded by DNA.

It is important to note that the use of RNA to encode transposases needs to be optimized for that specific system. It is not possible to compare results from independently published studies due asynchronous variables such as different transposase species, capping methodologies, poly-A tail lengths, cis-acting untranslated sequences, and transfection methods. To dampen the risk of insertional mutagenesis associated to genetic modification of cells *via* chromosomal integration, the choice of the vector system should be taken in account to avoid insertion into proto-oncogenes or transcription start sites that can lead to unintended transformation *via* insertional mutagenesis. We opted for SB system on the basis of the safer, more random integration pattern, as it does not demonstrate preferences to insert the transgene in active genes or TSSs thereby lowering the probability of integration in oncogenic genes. As previously highlighted in 4.3 paragraph, it was recently reported that 2 of 10 patients treated with anti-CD19 CAR T generated with the PB transposase system developed CAR-expressing CD4+ T cell lymphoma (132). To our knowledge, there have been no reports of insertional mutagenesis using the SB11 or SB100X transposase systems.

### 5.3 Clinical Applications

Potential safety advantages of transient CAR expression from mRNA may offer lower toxicity in both hematologic and solid tumor settings, especially outside of B-cell malignancies where off-tumor on-target collateral damage to healthy cells is a concern. Early phase clinical studies were conducted in hematological malignancies targeting CD123 and CD19 and in solid tumors targeting mesothelin and c-Met [reviewed in **Table 3** (165)].

While the studies report to be safe and generally lacking serious adverse events, one common denominator was the requirement for repeated dosing with 3-6 high doses. The need for multiple infusions of high doses of mRNA CAR T cells is most likely related to the lack of genetically modified cell persistence and aims to increase the duration of *in vivo* activity in these patients but repeat dosing may lead to other complications. Maus et al. reported a case of severe anaphylactic shock in a patient due to repeated doses of mesothelin-targeted CAR T cells, probably due to the murine origin of the single-chain fragment variable (173). In addition, difficulties in producing enough mRNA CD123 CAR T product to sustain multiple doses have been reported, with only 60% of the planned T cell doses being able to be successfully produced, questioning about the feasibility of obtaining such a large starting material from patients. Given the lack of anti-leukemic efficacy, the trial was terminated, raising concerns about the efficacy of transient approaches to eradicate proliferating diseases. However, the study was able to confirm the safety of the approach, and thus proceed to clinical trials with a stable transfer approach using CAR T cells transduced with lentivirus (174).

## 6 Electroporation, Hybrid Viral-Transposon Vectors and Nanocarriers

### 6.1 Electroporation

Transfection or electroporation methods are typically used to deliver the mRNA and transposon vectors into cells. Indeed, nucleic acids are not able to penetrate spontaneously in target cells as viral vectors do through infection. To facilitate nucleic acid entry into cells, cells suspended in an electroporation cuvette are subjected to an electric field determined by a suitable electrical pulse. The process generate temporary pores in the cell membrane that allow vector penetration, and then seal up once the electric field is withdrawn. Once the nucleic acids enter the cell, they efficiently migrate into the nucleus. The electroporation efficiency depends on the voltage, the number of pulses, pulse width, and on the cell type and activation state. Furthermore, temperature, electroporation buffer, DNA and cell concentration influence the transduction efficiency. High-intensity pulses generally result in higher transduction efficiency but affect cellular viability. Small-scale electroporation can be achieved using Nucleofector 4D (Lonza, Basel, Switzerland) Neon (Thermo Fisher Scientific, Waltham) which use a cuvette and a pipette tip chamber, respectively. Other commercialized instruments are for example the Celetrix electroporation system (Celetrix, Manassas, VA, USA), and the BTX ECM 830 system (Harvard Bioscience, Hollistone USA). Commercially available electroporation devices for large-scale electroporation are Lonza LV unit and Maxcyte GTx (MaxCyte, Gaithersburg, MD, USA) platform. Lonza LV unit allows for closed electroporation of 1x10^7 to 1x10^9 cells. Maxcyte GTx device is a GMP-compliant, clinical-grade instrument and can electroporate up to 20x10^9 cells using flow electroporation technology.

### 6.2 Hybrid Viral-Transposon Vectors

Hybrid viral-transposon vector combine the entry properties of viral vectors with the integrative characteristic of transposons. This is particularly convenient when using recombinant adenovirus (Ad), a common vector due to its broad tropism, large carrying capacity, and optimal efficient transduction regardless the mitotic status of target cells. Ad has non-integrative features and thus results in transient transgene expression. The addition of integrative elements into the viral genome could overcome this limitation. A recombinant Ad vector containing a PB-transposon was shown to allow the integration of the transgene into the genome in presence of PB-transposase, included in the vector design or co-delivered. With these methods, stable expression of a reporter transgene was achieved in 20-40% of mouse liver cells after infusion and lasts for at least 5 months (175). Similarly, various groups have combined AAV vectors with the PB transposon system for *in vivo* delivery to correct several diseases such as diabetes type 1 (176), cystic fibrosis (177), and others. Recombinant adeno-associated viral vectors (rAAVs), Herpes simplex virus type-1 (HSV) vectors, baculovirus expression vectors (BEVs) have been tested with the SB transposon system (178).

### 6.3 *In Vivo* CAR T Cell Generation and Nanocarriers

As previously discussed, most protocols for adoptive T cell therapies require the collection of T cells and their *ex vivo* genetic manipulation. Patients are connected to an apheresis machine for several hours to extract T cells. Manufacturing involves activating and transducing purified T cells, expanding them *in vitro* for approximately 2 weeks and finally washing and concentrating them prior to administration. Often cells have to be cryopreserved in a central facility and transported to remote treatment centers. Quality controls on final product are mandatory for each batch. Manufacturing must be conducted under GMP conditions, and the entire process is expensive and needs specific resources, facilities and economic capital. In addition, because most CAR T products are currently obtained from an autologous source and thus from the patient’s own cells, there are no economies of scale. In order to overcome the complexity of *ex vivo* manufacturing, *in vivo* CAR T cell generation is emerging as a new prospect and exploits the use of liposomal formulations, nanoparticles (NP), cell-penetrating peptides or advanced electroporation methods (179).

In this context, there are many promising attempts using nanocarriers composed of polymeric or lipid nanoparticles to produce CAR T cells directly from the patient’s circulating T cells. Nanocarriers composed of biodegradable polymers are coated with ligands that targets them to specific cells and can encapsulate different substances such as drugs or non-viral transgenes. Smith and collaborators loaded nanoparticles with a PB transposon/transposase system encoding CAR (180). To ensure the specific delivery of the gene cargo to T cells, they coupled T cell targeting anti-CD3ef(ab’)2 fragments to the surface of biodegradable poly (beta-amino ester) NPs. They co-encapsulated two PB plasmids, encoding a murine anti-CD19 (m194-1BBz) CAR and a hyperactive form of the PB transposase (iPB7), respectively, into the polymeric nanocarriers. DNA-carrying NPs were able to efficiently introduce the CAR genes into T cell nuclei, bind circulating T cells and cause tumor regression in mice with similar efficacies to adoptive T cell therapy. Although *in situ* programming of CAR T cells through injectable polymeric NPs is possible, this strategy has some limitations such as NP loading capacity which difficultly fits the large size of plasmids and the need to codelivery the transposase vector. Moreover, as soon as NPs are infused, the small number of *in situ* transfected CAR T cells needs antigen drive expansion to show a visible anti-tumor activity. For these reasons, the same group evaluated the use of *in vitro* transcribed (IVT) mRNA encoding disease specific CAR or TCR encapsulated in poly (beta-amino ester) NPs, coupled to anti-CD8 antibody for specific T cell delivery. IVT-mRNA has the advantages of being directly translated into therapeutic proteins, without the need to enter the nucleus, improving transfection rates and avoiding uncontrolled insertional mutations and promoter dependence. Using this technology, circulating T cells have been reprogrammed with leukemia specific CAR and showed an anti-tumor efficacy when NPs were provided with repetitive infusion (181).

**Figure 4:**
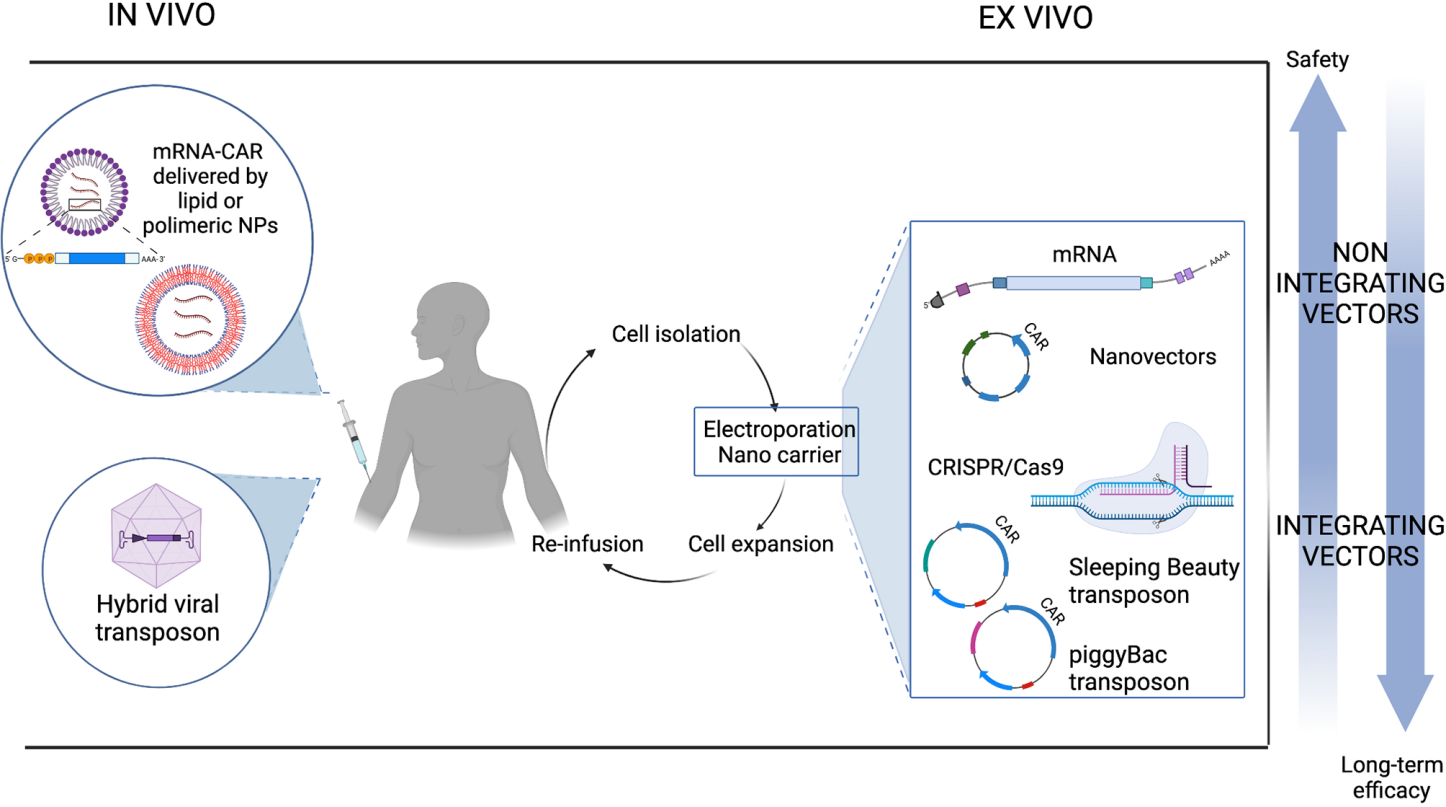
Non-viral CAR T cell generation.

Building on the dazzling success of the application of lipid NPs encapsulated mRNA (LNP-mRNA) vaccines formulated against SARS-CoV-2, the study by Rurik et al. provides a great proof of concept on the possibility of producing CAR T *in vivo* for the treatment of cardiac injury (182). By employing CD5-targeted NPs, they succeeded in delivering the nanoparticles into T lymphocytes to generate CAR T *in vitro* and *in vivo*. *In vitro*, this strategy can drive the expression of an anti-FAP CAR efficiently (83% of cells expressing CAR measured by flow cytometry) and transiently, resulting in a dose-dependent killing activity similar to virally engineered cells. Administration of CD5-targeted LNP showed a reduction in fibrosis and restoration of cardiac function in a syngeneic model of cardiac injury, proving their ability to reprogram T cells *in vivo*. Although this platform is not suitable for diseases that require complete elimination of pathological cells, such as some forms of cancer, undoubtedly, for other applications, the ability to generate CAR T *in vivo* and the inherently transient nature of mRNA have the advantage of limiting toxicities, titrating doses, and offering an off-the-shelf process. **Figure 4** illustrates how non-viral CAR T cells can be generated through *in vivo* and *ex vivo* transfection.

## 7 Non-viral CAR T Cell Therapy: The Future

### 7.1 Nanovectors and Combination With Gene-Editing

To address future challenges, in addition to transposon platforms, an additional non-viral tool for gene engineering is represented by nanovectors whose use is gaining ground as a possible solution to overcome current barriers in gene delivery such as toxicity and low transfection efficiency. Among the latest advances in nanotechnology [reviewed in (183)], one of the most cutting-edge finding is reported by Bozza and collaborators, who developed a non-integrating DNA nanovector with the ability to generate CAR T cells that are active both *in vitro* and *in vivo*. This platform contains no viral components and is capable of replicating extra-chromosomally in the nucleus of dividing cells, leading to persistent transgene expression, in the absence of integration and consequently genotoxicity. Moreover, it also shares all the advantages of non-viral vectors: it seems to be non-immunogenic, easy, simple, versatile and affordable to produce (184).

Also on the manufacturing front, progress is being made through the employment of biomaterials developed to improve isolation, activation, and genetic modifications of CAR T cells [reviewed in (185)]. One example is the use of a synthetic DNA aptamer and complementary reversal agent technology that permit isolation of label-free CD8+ T cells with high purity and yield from PBMCs. The great advantage of this method is represented by the possibility to isolate multiple distinct T cell populations in a single isolation step through aptamers with different specificities. Biomaterials, such as polymers and particles are also emerging to facilitate the T-cell activation process by eliminating the bead removal step, the first example of which is Miltenyi’s T Cell TransAct. Another area in which substantial investment is underway is the ability to make the CAR T manufacturing process scalable, through first of all automation and the use of closed systems, but also the use of allogeneic products, as reviewed by Abou-el-Enein and colleagues (186). To this end, our lab has applied CAR T cells manufactured in-house from allo-transplant donors in patients previously transplanted with HSCT. The proven safety and therapeutic efficacy of this approach represents a proof of concept towards an off-the-shelf product, and in this perspective, we are currently working on the design of a study that will employ haploidentical allogeneic cells outside the transplantation context.

In the field of integrative non-viral approach, site-specific insertion approaches are undergoing rapid technological advances, thanks also to the ease of use of systems such as CRISPR/Cas9, which have also led to early clinical developments in the field of immunotherapy (187). Interestingly, it has recently been reported that electroporation of Ribonucleoprotein (RNP) and linear double-strand long DNA (>1kb) template reduces the toxicity of double-strand linear template, a finding that validates CRISPR/Cas9 as part of the state-of the-art in virus-free genome engineering technologies. Yet, in this early study, the integration efficiency was around 10% when applied to insertion loads of 1500 bp, as in the case of replacing the endogenous TCR with the antigen-specific TCR 1G4 NY-ESO-1 (188). Gene editing and targeted knock-in (KI) depend on the processes of host DNA double-strand break (DSB) repair and homology-directed repair (HDR), respectively, and HDR generally occurs with low frequency in primary cells and is restricted to small transgenes. Despite the low transfection efficiency due to the above intrinsic properties, there has been quite some progress for non-viral gene editing in adoptive T cell therapy, particularly in TCR engineering. Compared to the other technologies described above, gene editing combined with targeted KI allows TCR replacement with concomitant removal of the endogenous TCR, resulting in physiological expression of the transgenic TCR through the endogenous promoter. When combined with the elimination of both the α- and β-chains, it allows physiological TCR expression in absence of chain mispairing. Isolation of KI cells and *in vitro* expansion allow to reach highly purified cells to cope with the low efficiency of KI (189). In a direct comparison with conventional editing by viral transduction, orthotopic TCR replacement (OTR) using a library of 51 CMV-specific TCRs was characterized by more homogeneous and physiological TCR transcription, while surface expression by viral vectors was influenced by transgene copy number, leading to more variable TCR expression, with impact on *in vitro* and *in vivo* functionality (190).

Even under optimized condition, some cellular toxicity still limits the application of non-viral CRISPS/Cas9 and appears to depend on the electroporation of double strand DNA (dsDNA) and RNP aggregates into cells and the endogenous immune response triggered by innate DNA sensor protein pathway. To address the first challenge, Nguyen D. N. et al. used poly-L-glutamic acid, which physically disperse large RNP aggregates into smaller complexes. Additionally, they implemented the incorporation of a truncated Cas9 target sequence (tCTS) at the end of homology arms in order to facilitate the shuttling of the template into the nucleus. The combination of the two systems resulted in high KI efficiency of up to 50% in a wide set of primary human hematopoietic cells (191). To address the endogenous immune response, inhibition of the DNA sensor protein pathway was proposed in combination with insertion of a 2015 bp long CAR into the TRAC locus of human T cells *via* CRISPR/Cas9-mediated HDR. In conjunction with the poly-L-glutamic acid nanoparticle strategy, the use of DNA-sensor inhibitors and HDR enhancers achieved high editing efficiency with a insertion rate of 68% (192). Two other recent works have used the CRISPR/Cas9 platform to integrate a CAR with promising results. By targeting an anti-GD2 CAR into the first exon of TRAC locus, an average KI efficiency of 15% was obtained, improved up to 45% by increasing the length of the homology arms on both sides of the CAR with final DNA template size of 3,4 Kb and resulting in 34% CAR positive cells (193). Moreover, by using a hybrid single strand (ss) DNA HDR template incorporating CTS sites, increased KI was obtained, up to 40% efficiency in combination with small molecule inhibitors (194).

To provide DNA insertion in the absence of DSB and HDR, CRISPR/Cas9 has recently been combined with transposons to increase the efficiency of RNA-guided integration, using a transposase protein to catalyze the integration (195, 196). Recently, experiments have also been conducted to combine CRISPR/Cas9 with SB transposon. The transposase protein was fused to a catalytically inactive Cas9 for providing single guide RNA-dependent DNA insertion in the absence of DSB and HDR, leading to enrichment of integrations near the sgRNA targets (197). Recent results obtained with gene editing techniques demonstrate their suitability for future non-viral clinical applications. Approaches to identify and reduce the risk of genomic rearrangements and translocations may obviate concerns associated with the collateral damage of the technique and thus allow for greater development of potential clinical applications of gene editing in immunotherapy.

## 8 Conclusion and Practical Considerations for Future Trials

Successful CAR T cell therapies are so far associated with T cells engineered with viral vectors. Yet, the emergence of relapse, the complexity of the manufacturing process, and the application of these technologies to other diseases including solid tumors, require a more complex design and gene transfer technology that fits these challenges. So much progress is being made with non-viral technologies to fill these gaps, and it is certain that a great step forward will be taken soon.

Previously discussed non-viral manufacturing methods are briefly summarize in **Table 4**.

**Table 4:** Summary of principal characteristics of methods in CAR T manufacturing.

	Characteristics and peculiarities	Pros/Cons	Technical requirement	Impact of costs
**Viral vectors**	Gammaretroviral vectors: Infection only in cycling cells Integration near TSS Lentiviral vectors: Infection in cycling/non cycling cells Integration in transcriptional regulatory region	Pros: Stable transduction Long term expression Cons: Limiting insert size Difficulty to scale synthesis up Risk of insertional oncogenesis More immunogenicity	Biosafety Level 2 Trained staff Cryopreserved facility	High costs Limited number of available manufacturing facilities globally, and lot size limitations
**Sleeping Beauty**	Tc1/mariner DNA Class II TE Cut-and-paste mechanism of insertion Transposon/transposase system Transfection in pre-activated and resting primary cells Close to random integration TA dinucleotide as target site CAG footprint	Pros: Easy to scale synthesis up Large cargo size (up to 100 kb BACs) Versatility Low immunogenicity Cons: Toxicity related to transduction procedure	Electroporation Cryopreserved facility	Relatively low cost Easier manufacturing process
**Piggy Bac**	PB superfamily DNA Class II TE Cut-and-paste mechanism of insertion Transposon/transposase system Transfection in pre-activated and resting primary cells Preference of integration near TSSs, CpG islands and DNase I hypersensitive sites TTAA dinucleotide as target site No footprint	Pros: Easy to scale synthesis up Large cargo size (200 kb BACs) Versatility Low immunogenicity Higher transposition activity Cons: High risk of gene dysregulation Toxicity related to transduction procedure	Electroporation Cryopreserved facility	Relatively low cost Easier manufacturing process
**mRNA**	Absence of integration Transient transfection	Pros: Availability of protocols for clinics Versatility and flexibility Safety transient expression (SB100X and PB transposase) Cons (mRNA encoding CAR): Short term potency Need for multiple doses	Electroporation Cation lipids Cationic polymers	High doses of mRNA CAR T are required to achieve efficacy
**Nanotechnology**	Nanocarriers or lipid nanoparticles coated with ligands ensure encapsulation of non-viral transgenes Ability to reprogram T cells *in vivo*	Pros: Low toxicity Off -the-shelf process Cons: Limited cargo capacity	Devices for scale up production Specialized staff	Costs of nanoparticles production and costs of encapsulated material

Non-viral CAR T cells engineered with transposon vectors have already shown some efficacy in early-stage clinical trials, but whether such systems may also show unexpected toxicities and comparable clinical outcomes remains to be seen in additional clinical studies. For first-in-human clinical trials, certain precautions have been implemented to ensure safety given the novelty of the gene transfer method. In our study (43), given the low-risk classification associated with non-viral vectors, physical containment of the vector and transfected cell product was managed at biosafety level 1. The duration of *in vitro* cell culture ensured the absence of plasmids at the end of culture. Whether the bacterial sequences below the limits of detection is sufficient to protect from an immune response by the host against bacterial sequences, however, is currently unknown. The mean transgene copy number was set at 5 as an upper limit, in order to limit the potential risk of genotoxicity. Others are instead using 8 as release criteria for transposon-generated CAR T cell products. Despite the almost random insertion profile of the transgene into the T cell genome, the study of integrations ensured the monitoring of the clonality of the CAR T population post-infusion. Based on this clinical experience, we therefore recommend the use of vectors and the development of manufacturing platforms designed to contain transposase activity and to limit the number of integrations, and the implementation of a detailed follow-up plan for clonality monitoring by integration site analysis.

We believe that the main takeaway from the efforts described in this review is the possibility of using novel non-viral engineering approaches in future clinical trials. In addition to non-viral integrating methods, we think that the use of episomal nanovectors may be advantageous for the *ex vivo* production of safer transfected cells. In addition, viral-transposon hybrid vectors and mRNA delivered by polymeric and lipid nanoparticles represent technological platforms that can generate stable or transient CAR T cells *in vivo*. We are on a learning curve and looking forward to seeing the results of these technologies in future clinical trials.

## Authors Contributions

CM conceptualized, wrote, and edited the manuscript. MP, AM, and IT wrote the manuscript. MP and AM designed the figures and prepared the tables. CN and AB edited the manuscript. All authors contributed to the article and approved the submitted version.

## Funding

CM has received funding from Ministero della Salute, grant number GR-2016-02363491 and Fonds zur förderung des akademischen nachwuchses (FAN) of the University of Zurich (UZH). AB and CM have received funding from AIRC/Cancer Research UK (CRUK)/Spanish Association Against Cancer Scientific Foundation (FC AECC), grant number 22791 (Accelerator award).

## Conflict of Interest

Authors CM and AB are inventors on a patent related to non-viral CAR-T cell therapy (European patent application 15801344), filed by the Tettamanti Foundation, and licensed to CoImmune. Authors CN and IT are executive officers of CoImmune. Author AB has received fundings from AIRC 5x1000 2018-21147, PRIN 2017-NAZ-0412, and the Ministero della Salute CAR-T project of the “Rete Oncologica”.

The remaining authors declare that the research was conducted in the absence of any commercial or financial conflict of interests.

## Publisher’s Note

All claims expressed in this article are solely those of the authors and do not necessarily represent those of their affiliated organizations, or those of the publisher, the editors and the reviewers. Any product that may be evaluated in this article, or claim that may be made by its manufacturer, is not guaranteed or endorsed by the publisher.

## References

[1] Eshhar Z Waks T Gross G Schindler DG. Specific Activation and Targeting of Cytotoxic Lymphocytes Through Chimeric Single Chains Consisting of Antibody-Binding Domains and the Gamma or Zeta Subunits of the Immunoglobulin and T-Cell Receptors. Proc Natl Acad Sci USA (1993) 90:720–4. doi: 10.1073/pnas.90.2.720 PMC45737 8421711

[2] Majzner RG Mackall CL. Clinical Lessons Learned From the First Leg of the CAR T Cell Journey. Nat Med (2019) 25:1341–55. doi: 10.1038/s41591-019-0564-6 31501612

[3] Maude SL Laetsch TW Buechner J Rives S Boyer M Bittencourt H. Tisagenlecleucel in Children and Young Adults With B-Cell Lymphoblastic Leukemia. N Engl J Med (2018) 378:439–48. doi: 10.1056/NEJMoa1709866 PMC5996391 29385370

[4] Gardner RA Finney O Annesley C Brakke H Summers C Leger K. Intent-To-Treat Leukemia Remission by CD19 CAR T Cells of Defined Formulation and Dose in Children and Young Adults. Blood (2017) 129:3322–31. doi: 10.1182/blood-2017-02-769208 PMC5482103 28408462

[5] Turtle CJ Hanafi L-A Berger C Gooley TA Cherian S Hudecek M. CD19 CAR-T Cells of Defined CD4+:CD8+ Composition in Adult B Cell ALL Patients. J Clin Invest (2016) 126:2123–38. doi: 10.1172/JCI85309 PMC4887159 27111235

[6] Park JH Rivière I Gonen M Wang X Sénéchal B Curran KJ. Long-Term Follow-Up of CD19 CAR Therapy in Acute Lymphoblastic Leukemia. N Engl J Med (2018) 378:449–59. doi: 10.1056/NEJMoa1709919 PMC6637939 29385376

[7] Curran KJ Margossian SP Kernan NA Silverman LB Williams DA Shukla N. Toxicity and Response After CD19-Specific CAR T-Cell Therapy in Pediatric/Young Adult Relapsed/Refractory B-ALL. Blood (2019) 134:2361–8. doi: 10.1182/blood.2019001641 PMC6933289 31650176

[8] Ghorashian S Kramer AM Onuoha S Wright G Bartram J Richardson R. Enhanced CAR T Cell Expansion and Prolonged Persistence in Pediatric Patients With ALL Treated With a Low-Affinity CD19 CAR. Nat Med (2019) 25:1408–14. doi: 10.1038/s41591-019-0549-5 31477906

[9] Turtle CJ Hanafi L-A Berger C Hudecek M Pender B Robinson E. Immunotherapy of non-Hodgkin’s Lymphoma With a Defined Ratio of CD8+ and CD4+ CD19-Specific Chimeric Antigen Receptor-Modified T Cells. Sci Transl Med (2016) 8:355ra116. doi: 10.1126/scitranslmed.aaf8621 PMC5045301 27605551

[10] Schuster SJ Bishop MR Tam CS Waller EK Borchmann P McGuirk JP. Tisagenlecleucel in Adult Relapsed or Refractory Diffuse Large B-Cell Lymphoma. N Engl J Med (2019) 380:45–56. doi: 10.1056/NEJMoa1804980 30501490

[11] Neelapu SS Locke FL Bartlett NL Lekakis LJ Miklos DB Jacobson CA. Axicabtagene Ciloleucel CAR T-Cell Therapy in Refractory Large B-Cell Lymphoma. N Engl J Med (2017) 377:2531–44. doi: 10.1056/NEJMoa1707447 PMC5882485 29226797

[12] Fry TJ Shah NN Orentas RJ Stetler-Stevenson M Yuan CM Ramakrishna S. CD22-Targeted CAR T Cells Induce Remission in B-ALL That Is Naive or Resistant to CD19-Targeted CAR Immunotherapy. Nat Med (2018) 24:20–8. doi: 10.1038/nm.4441 PMC5774642 29155426

[13] Brudno JN Maric I Hartman SD Rose JJ Wang M Lam N. T Cells Genetically Modified to Express an Anti-B-Cell Maturation Antigen Chimeric Antigen Receptor Cause Remissions of Poor-Prognosis Relapsed Multiple Myeloma. J Clin Oncol Off J Am Soc Clin Oncol (2018) 36:2267–80. doi: 10.1200/JCO.2018.77.8084 PMC6067798 29812997

[14] Raje N Berdeja J Lin Y Siegel D Jagannath S Madduri D. Anti-BCMA CAR T-Cell Therapy Bb2121 in Relapsed or Refractory Multiple Myeloma. N Engl J Med (2019) 380:1726–37. doi: 10.1056/NEJMoa1817226 PMC8202968 31042825

[15] Pasquini MC Hu Z-H Curran K Laetsch T Locke F Rouce R. Real-World Evidence of Tisagenlecleucel for Pediatric Acute Lymphoblastic Leukemia and Non-Hodgkin Lymphoma. Blood Adv (2020) 4:5414–24. doi: 10.1182/bloodadvances.2020003092 PMC7656920 33147337

[16] Nastoupil LJ Jain MD Feng L Spiegel JY Ghobadi A Lin Y. Standard-Of-Care Axicabtagene Ciloleucel for Relapsed or Refractory Large B-Cell Lymphoma: Results From the US Lymphoma CAR T Consortium. J Clin Oncol Off J Am Soc Clin Oncol (2020) 38:3119–28. doi: 10.1200/JCO.19.02104 PMC7499611 32401634

[17] Abramson JS Palomba ML Gordon LI Lunning MA Wang M Arnason J. Lisocabtagene Maraleucel for Patients With Relapsed or Refractory Large B-Cell Lymphomas (TRANSCEND NHL 001): A Multicentre Seamless Design Study. Lancet (Lond Engl) (2020) 396:839–52. doi: 10.1016/S0140-6736(20)31366-0 32888407

[18] Strati P Ahmed S Furqan F Fayad LE Lee HJ Iyer SP. Prognostic Impact of Corticosteroids on Efficacy of Chimeric Antigen Receptor T-Cell Therapy in Large B-Cell Lymphoma. Blood (2021) 137:3272–6. doi: 10.1182/blood.2020008865 PMC8351896 33534891

[19] Jacobson C Chavez JC Sehgal AR William BM Munoz J Salles G. Primary Analysis of Zuma-5: A Phase 2 Study of Axicabtagene Ciloleucel (Axi-Cel) in Patients With Relapsed/Refractory (R/R) Indolent Non-Hodgkin Lymphoma (iNHL). Blood (2020) 136:40–1. doi: 10.1182/blood-2020-136834

[20] Munshi NC Anderson LDJ Shah N Madduri D Berdeja J Lonial S. Idecabtagene Vicleucel in Relapsed and Refractory Multiple Myeloma. N Engl J Med (2021) 384:705–16. doi: 10.1056/NEJMoa2024850 33626253

[21] Gill S Brudno JN. CAR T-Cell Therapy in Hematologic Malignancies: Clinical Role, Toxicity, and Unanswered Questions. Am Soc Clin Oncol Educ B (2021) 41:1–20. doi: 10.1200/EDBK_320085 33989023

[22] Roselli E Faramand R Davila ML. Insight Into Next-Generation CAR Therapeutics: Designing CAR T Cells to Improve Clinical Outcomes. J Clin Invest (2021) 131(2):e142030. doi: 10.1172/JCI142030 PMC7810492 33463538

[23] Scholler J Brady TL Gwendolyn Binder-Scholl W-TH Plesa G Hege KM Vogel AN. Decade-Long Safety and Function of Retroviral-Modified Chimeric Antigen Receptor T-Cells. Sci Transl Med (2015) 34:132ra53. doi: 10.1126/scitranslmed.3003761.Decade-Long PMC4368443 22553251

[24] Bulcha JT Wang Y Ma H Tai PWL Gao G. Viral Vector Platforms Within the Gene Therapy Landscape. Signal Transduct Target Ther (2021) 6:53. doi: 10.1038/s41392-021-00487-6 33558455 PMC7868676

[25] Kamdar M Solomon SR Arnason JE Johnston PB Glass B Bachanova V. Lisocabtagene Maraleucel (Liso-Cel), a CD19-Directed Chimeric Antigen Receptor (CAR) T Cell Therapy, Versus Standard of Care (SOC) With Salvage Chemotherapy (CT) Followed By Autologous Stem Cell Transplantation (ASCT) As Second-Line (2l) Treatment in Pati. Blood (2021) 138:91. doi: 10.1182/blood-2021-147913 33881503

[26] Neelapu SS Locke FL Bartlett NL Lekakis LJ Reagan PM Miklos DB. Comparison of 2-Year Outcomes With CAR T Cells (ZUMA-1) vs Salvage Chemotherapy in Refractory Large B-Cell Lymphoma. Blood Adv (2021) 5:4149–55. doi: 10.1182/bloodadvances.2020003848 PMC8945634 34478487

[27] Schröder ARW Shinn P Chen H Berry C Ecker JR Bushman F. HIV-1 Integration in the Human Genome Favors Active Genes and Local Hotspots. Cell (2002) 110:521–9. doi: 10.1016/s0092-8674(02)00864-4 12202041

[28] Wu X Li Y Crise B Burgess SM. Transcription Start Regions in the Human Genome are Favored Targets for MLV Integration. Science (2003) 300:1749–51. doi: 10.1126/science.1083413 12805549

[29] Wang GP Levine BL Binder GK Berry CC Malani N McGarrity G. Analysis of Lentiviral Vector Integration in HIV+ Study Subjects Receiving Autologous Infusions of Gene Modified CD4+ T Cells. Mol Ther (2009) 17:844–50. doi: 10.1038/mt.2009.16 PMC2835137 19259065

[30] Hacein-Bey-Abina S Garrigue A Wang GP Soulier J Lim A Morillon E. Insertional Oncogenesis in 4 Patients After Retrovirus-Mediated Gene Therapy of SCID-X1. J Clin Invest (2008) 118:3132–42. doi: 10.1172/JCI35700 PMC2496963 18688285

[31] Hacein-Bey-Abina S Von Kalle C Schmidt M McCormack MP Wulffraat N Leboulch P. LMO2-Associated Clonal T Cell Proliferation in Two Patients After Gene Therapy for SCID-X1. Science (2003) 302:415–9. doi: 10.1126/science.1088547 14564000

[32] Shah NN Qin H Yates B Su L Shalabi H Raffeld M. Clonal Expansion of CAR T Cells Harboring Lentivector Integration in the CBL Gene Following Anti-CD22 CAR T-Cell Therapy. Blood Adv (2019) 3:2317–22. doi: 10.1182/bloodadvances.2019000219 PMC6693002 31387880

[33] Fraietta JA Nobles CL Sammons MA Lundh S Carty SA Reich TJ. Disruption of TET2 Promotes the Therapeutic Efficacy of CD19-Targeted T Cells. Nature (2018) 558:307–12. doi: 10.1038/s41586-018-0178-z PMC6320248 29849141

[34] Milone MC O’Doherty U. Clinical Use of Lentiviral Vectors. Leukemia (2018) 32:1529–41. doi: 10.1038/s41375-018-0106-0 PMC6035154 29654266

[35] U.S Food and Drug Administration. Testing of Retroviral Vector-Based Human Gene Therapy Products for Replication Competent Retrovirus During Product Manufacture and Patient Follow-Up - Draft Guidance for Industry. FDA (2018) 16. Docket number: FDA-1999-D-0081

[36] European Medicine Agency. Committee for Medicinal Products for Human Use (Chmp) Guideline on Development and Manufacture of Lentiviral Vectors. Reproduction (2005).

[37] Lamers CHJ Willemsen R van Elzakker P van Steenbergen-Langeveld S Broertjes M Oosterwijk-Wakka J. Immune Responses to Transgene and Retroviral Vector in Patients Treated With Ex Vivo-Engineered T Cells. Blood (2011) 117:72–82. doi: 10.1182/blood-2010-07-294520 20889925

[38] McClintock B. The Origin and Behavior of Mutable Loci in Maize. Proc Natl Acad Sci (1950) 36:344 LP–355. doi: 10.1073/pnas.36.6.344 15430309 PMC1063197

[39] SanMiguel P Tikhonov A Jin YK Motchoulskaia N Zakharov D Melake-Berhan A. Nested Retrotransposons in the Intergenic Regions of the Maize Genome. Science (1996) 274:765–8. doi: 10.1126/science.274.5288.765 8864112

[40] Feschotte C Pritham EJ. DNA Transposons and the Evolution of Eukaryotic Genomes. Annu Rev Genet (2007) 41:331–68. doi: 10.1146/annurev.genet.40.110405.090448 PMC2167627 18076328

[41] Carmona LM Schatz DG. New Insights Into the Evolutionary Origins of the Recombination-Activating Gene Proteins and V(D)J Recombination. FEBS J (2017) 284:1590–605. doi: 10.1111/febs.13990 PMC5459667 27973733

[42] Bordenstein SR Reznikoff WS. Mobile DNA in Obligate Intracellular Bacteria. Nat Rev Microbiol (2005) 3:688–99. doi: 10.1038/nrmicro1233 16138097

[43] Magnani CF Gaipa G Lussana F Belotti D Gritti G Napolitano S. Sleeping Beauty-Engineered CAR T Cells Achieve Antileukemic Activity Without Severe Toxicities. J Clin Invest (2020) 130:6021–33. doi: 10.1172/JCI138473 PMC7598053 32780725

[44] Magnani CF Mezzanotte C Cappuzzello C Bardini M Tettamanti S Fazio G. Preclinical Efficacy and Safety of CD19CAR Cytokine-Induced Killer Cells Transfected With Sleeping Beauty Transposon for the Treatment of Acute Lymphoblastic Leukemia. Hum Gene Ther (2018) 29:602–13. doi: 10.1089/hum.2017.207 29641322

[45] Gogol-Döring A Ammar I Gupta S Bunse M Miskey C Chen W. Genome-Wide Profiling Reveals Remarkable Parallels Between Insertion Site Selection Properties of the MLV Retrovirus and the Piggybac Transposon in Primary Human CD4+ T Cells. Mol Ther (2016) 24:592–606. doi: 10.1038/mt.2016.11 26755332 PMC4786924

[46] Henssen AG Henaff E Jiang E Eisenberg AR Carson JR Villasante CM. Genomic DNA Transposition Induced by Human PGBD5. Elife (2015) 4:e10565. doi: 10.7554/eLife.10565 26406119 PMC4625184

[47] Ivics Z. Endogenous Transposase Source in Human Cells Mobilizes Piggybac Transposons. Mol Ther (2016) 24:851–4. doi: 10.1038/mt.2016.76 PMC4881781 27198853

[48] Kowalski PS Rudra A Miao L Anderson DG. Delivering the Messenger: Advances in Technologies for Therapeutic mRNA Delivery. Mol Ther (2019) 27:710–28. doi: 10.1016/j.ymthe.2019.02.012 PMC6453548 30846391

[49] Ritchie H Mathieu E Ortiz-Ospina E Roser M Hasell J Appel C. Coronavirus Pandemic (COVID-19). (2020).

[50] Mathieu E Ritchie H Ortiz-Ospina E Roser M Hasell J Appel C. A Global Database of COVID-19 Vaccinations. Nat Hum Behav (2021) 5:947–53. doi: 10.1038/s41562-021-01122-8 33972767

[51] Manfredi F Cianciotti BC Potenza A Tassi E Noviello M Biondi A. TCR Redirected T Cells for Cancer Treatment: Achievements, Hurdles, and Goals. Front Immunol (2020) 11:1689. doi: 10.3389/fimmu.2020.01689 33013822 PMC7494743

[52] Ivics Z Hackett PB Plasterk RH Izsvák Z. Molecular Reconstruction of Sleeping Beauty, a Tc1-Like Transposon From Fish, and Its Transposition in Human Cells. Cell (1997) 91:501–10. doi: 10.1016/S0092-8674(00)80436-5 9390559

[53] Ivics Z Izsvák Z. The Expanding Universe of Transposon Technologies for Gene and Cell Engineering. Mobile DNA (2010) 1:25. doi: 10.1186/1759-8753-1-25 21138556 PMC3016246

[54] Ivics Z Izsvák Z. Nonviral Gene Delivery With the Sleeping Beauty Transposon System. Hum Gene Ther (2011) 22:1043–51. doi: 10.1089/hum.2011.143 21867398

[55] Ivics Z Li MA Mátés L Boeke JD Nagy A Bradley A. Transposon-Mediated Genome Manipulation in Vertebrates. Nat Methods (2009) 6:415–22. doi: 10.1038/nmeth.1332 PMC2867038 19478801

[56] Izsvák Z Hackett PB Cooper LJN Ivics Z. Translating Sleeping Beauty Transposition Into Cellular Therapies: Victories and Challenges. BioEssays (2010) 32:756–67. doi: 10.1002/bies.201000027 PMC3971908 20652893

[57] Hackett PB Largaespada DA Cooper LJN. and Transposase System for Human Application. Mol Ther (2010) 18:674–83. doi: 10.1038/mt.2010.2 PMC2862530 20104209

[58] Hackett PB Jr. Aronovich EL Hunter D Urness M Bell JB Kass SJ. Efficacy and Safety of Sleeping Beauty Transposon-Mediated Gene Transfer in Preclinical Animal Studies. Curr Gene Ther (2011) 11:341–9. doi: 10.2174/156652311797415827 PMC3728161 21888621

[59] Boehme P Doerner J Solanki M Jing L Zhang W Ehrhardt A. The Sleeping Beauty Transposon Vector System for Treatment of Rare Genetic Diseases: An Unrealized Hope? Curr Gene Ther (2015) 15:255–65. doi: 10.2174/1566523215666150126121353 25619886

[60] Narayanavari SA Chilkunda SS Izsv Ak Z. Critical Reviews in Biochemistry and Molecular Biology Sleeping Beauty Transposition: From Biology to Applications. Biochem Mol Biol (2016) 52:18–44. doi: 10.1080/10409238.2016.1237935 27696897

[61] VandenDriessche T Ivics Z Izsvák Z Chuah MKL. Emerging Potential of Transposons for Gene Therapy and Generation of Induced Pluripotent Stem Cells. Blood (2009) 114:1461–8. doi: 10.1182/blood-2009-04-210427 19471016

[62] Finnegan DJ. Eukaryotic Transposable Elements and Genome Evolution. Trends Genet (1989) 5:103–7. doi: 10.1016/0168-9525(89)90039-5 2543105

[63] Izsvák Z Khare D Behlke J Heinemann U Plasterk RH Ivics Z. Involvement of a Bifunctional, Paired-Like DNA-Binding Domain and a Transpositional Enhancer in Sleeping Beauty Transposition. J Biol Chem (2002) 277:34581–8. doi: 10.1074/jbc.M204001200 12082109

[64] Ammar I Izsvák Z Ivics Z. The Sleeping Beauty Transposon Toolbox. Methods Mol Biol (2012) 859:229–40. doi: 10.1007/978-1-61779-603-6_13 22367875

[65] Wang J DeClercq JJ Hayward SB Li PWL Shivak DA Gregory PD. Highly Efficient Homology-Driven Genome Editing in Human T Cells by Combining Zinc-Finger Nuclease mRNA and AAV6 Donor Delivery. Nucleic Acids Res (2016) 44(3):e30. doi: 10.1093/nar/gkv1121 26527725 PMC4756813

[66] Cui Z Geurts AM Liu G Kaufman CD Hackett PB. Structure–Function Analysis of the Inverted Terminal Repeats of the Sleeping Beauty Transposon. J Mol Biol (2002) 318:1221–35. doi: 10.1016/S0022-2836(02)00237-1 12083513

[67] Mátés L Chuah MKL Belay E Jerchow B Manoj N Acosta-Sanchez A. Molecular Evolution of a Novel Hyperactive Sleeping Beauty Transposase Enables Robust Stable Gene Transfer in Vertebrates. Nat Genet (2009) 41:753–61. doi: 10.1038/ng.343 19412179

[68] Jin Z Maiti S Huls H Singh H Olivares S Mátés L. The Hyperactive Sleeping Beauty Transposase SB100X Improves the Genetic Modification Of T Cells to Express a Chimeric Antigen Receptor. Gene Ther (2011) 18:849–56. doi: 10.1038/gt.2011.40 PMC4083583 21451576

[69] Voigt F Wiedemann L Zuliani C Querques I Sebe A Mátés L. Sleeping Beauty Transposase Structure Allows Rational Design of Hyperactive Variants for Genetic Engineering. Nat Commun (2016) 7:11126. doi: 10.1038/ncomms11126 27025571 PMC4820933

[70] Amberger M Ivics Z. Latest Advances for the Sleeping Beauty Transposon System: 23 Years of Insomnia But Prettier Than Ever: Refinement and Recent Innovations of the Sleeping Beauty Transposon System Enabling Novel, Nonviral Genetic Engineering Applications. BioEssays (2020) 42:1–14. doi: 10.1002/bies.202000136 32939778

[71] Querques I Mades A Zuliani C Miskey C Alb M Grueso E. A Highly Soluble Sleeping Beauty Transposase Improves Control of Gene Insertion. Nat Biotechnol (2019) 37:1502–12. doi: 10.1038/s41587-019-0291-z PMC6894935 31685959

[72] Vigdal T Kaufman C Izsvák Z Voytas D Ivics Z. Common Physical Properties of DNA Affecting Target Site Selection of Sleeping Beauty and Other Tc1/mariner Transposable Elements. J Mol Biol (2002) 323:441–52. doi: 10.1016/S0022-2836(02)00991-9 12381300

[73] Sandoval-Villegas N Nurieva W Amberger M Ivics Z. Contemporary Transposon Tools: A Review and Guide Through Mechanisms and Applications of Sleeping Beauty, Piggybac and Tol2 for Genome Engineering. Int J Mol Sci (2021) 22(10):5084. doi: 10.3390/ijms22105084 34064900 PMC8151067

[74] Huang X Guo H Tammana S Jung YC Mellgren E Bassi P. Gene Transfer Efficiency and Genome-Wide Integration Profiling of Sleeping Beauty, Tol2, and PiggyBac Transposons in Human Primary T Cells. Mol Ther (2010) 18:1803–13. doi: 10.1038/mt.2010.141 PMC2951558 20606646

[75] Izsvák Z Ivics Z Plasterk RH. Sleeping Beauty, a Wide Host-Range Transposon Vector for Genetic Transformation in Vertebrates. J Mol Biol (2000) 302:93–102. doi: 10.1006/jmbi.2000.4047 10964563

[76] Fischer SEJ van Luenen HGAM Plasterk RHA. Cis Requirements for Transposition of Tc1-Like Transposons in C. Elegans. Mol Gen Genet MGG (1999) 262:268–74. doi: 10.1007/PL00008641 10517322

[77] Zayed H Izsvák Z Walisco O Ivics Z. Development of Hyperactive Sleeping Beauty Transposon Vectors by Mutational Analysis. Mol Ther (2004) 9:292–304. doi: 10.1016/j.ymthe.2003.11.024 14759813

[78] Turchiano G Latella MC Döring AG Cattoglio C Mavilio F Izsvák Z. Correction: Genomic Analysis of Sleeping Beauty Transposon Integration in Human Somatic Cells. PloS One (2020) 15:e0228703. doi: 10.1371/journal.pone.0228703 31999797 PMC6992176

[79] Rostovskaya M Fu J Obst M Baer I Weidlich S Wang H. Transposon-Mediated BAC Transgenesis in Human ES Cells. Nucleic Acids Res (2012) 40:e150–0. doi: 10.1093/nar/gks643 PMC3479164 22753106

[80] Rostovskaya M Naumann R Fu J Obst M Mueller D Stewart AF. Transposon Mediated BAC Transgenesis *via* Pronuclear Injection of Mouse Zygotes. Genesis (2013) 51:135–41. doi: 10.1002/dvg.22362 23225373

[81] Monjezi R Miskey C Gogishvili T Schleef M Schmeer M Einsele H. Enhanced CAR T-Cell Engineering Using Non-Viral Sleeping Beauty Transposition From Minicircle Vectors. Leukemia (2017) 31:186–94. doi: 10.1038/leu.2016.180 27491640

[82] Shankar R Schmeer M Schleef M. Minicircles: Next-Generation Gene Vectors. Cell Gene Ther Insights (2017) 3:285–300. doi: 10.18609/cgti.2017.020

[83] Sharma N Cai Y Bak RO Jakobsen MR Schrøder LD Mikkelsen JG. Efficient Sleeping Beauty DNA Transposition From DNA Minicircles. Mol Ther Nucleic Acids (2013) 2:e74–4. doi: 10.1038/mtna.2013.1 PMC3586802 23443502

[84] Hodge R Narayanavari SA Izsvák Z Ivics Z. Wide Awake and Ready to Move: 20 Years of Non-Viral Therapeutic Genome Engineering With the Sleeping Beauty Transposon System. Hum Gene Ther (2017) 28:842–55. doi: 10.1089/hum.2017.130 28870121

[85] Huang X Wilber AC Bao L Tuong D Tolar J Orchard PJ. Stable Gene Transfer and Expression in Human Primary T Cells by the Sleeping Beauty Transposon System. Blood (2006) 107:483–91. doi: 10.1182/BLOOD-2005-05-2133 PMC1895607 16189271

[86] Singh H Manuri PR Olivares S Dara N Dawson MJ Huls H. Redirecting Specificity of T-Cell Populations For CD19 Using the Sleeping Beauty System. Cancer Res (2008) 68:2961–71. doi: 10.1158/0008-5472.CAN-07-5600 PMC2424272 18413766

[87] Magnani CF Turazzi N Benedicenti F Calabria A Tenderini E Tettamanti S. Immunotherapy of Acute Leukemia by Chimeric Antigen Receptor-Modified Lymphocytes Using an Improved Sleeping Beauty Transposon Platform. Oncotarget (2016) 7:51581–97. doi: 10.18632/oncotarget.9955 PMC5239498 27323395

[88] Introna M Lussana F Algarotti A Gotti E Valgardsdottir R Micò C. Phase II Study of Sequential Infusion of Donor Lymphocyte Infusion and Cytokine-Induced Killer Cells for Patients Relapsed After Allogeneic Hematopoietic Stem Cell Transplantation. Biol Blood Marrow Transplant J Am Soc Blood Marrow Transplant (2017) 23:2070–8. doi: 10.1016/j.bbmt.2017.07.005 28712935

[89] Introna M Borleri G Conti E Franceschetti M Barbui AM Broady R. Repeated Infusions of Donor-Derived Cytokine-Induced Killer Cells in Patients Relapsing After Allogeneic Stem Cell Transplantation: A Phase I Study. Haematologica (2007) 92:952–9. doi: 10.3324/haematol.11132 17606446

[90] Rambaldi A Biagi E Bonini C Biondi A Introna M. Cell-Based Strategies to Manage Leukemia Relapse: Efficacy and Feasibility of Immunotherapy Approaches. Leukemia (2015) 29:1–10. doi: 10.1038/leu.2014.189 24919807

[91] Schmeel FC Schmeel LC Gast S-M Schmidt-Wolf IGH. Adoptive Immunotherapy Strategies With Cytokine-Induced Killer (CIK) Cells in the Treatment of Hematological Malignancies. Int J Mol Sci (2014) 15:14632–48. doi: 10.3390/ijms150814632 PMC4159872 25196601

[92] Rotiroti MC Buracchi C Arcangeli S Galimberti S Valsecchi MG Perriello VM. Targeting CD33 in Chemoresistant AML Patient-Derived Xenografts by CAR-CIK Cells Modified With an Improved SB Transposon System. Mol Ther (2020) 28:1974–86. doi: 10.1016/j.ymthe.2020.05.021 PMC7474266 32526203

[93] Ghassemi S Durgin JS Nunez-Cruz S Patel J Leferovich J Pinzone M. Rapid Manufacturing of Non-Activated Potent CAR T Cells. Nat BioMed Eng (2022) 6:118–28. doi: 10.1038/s41551-021-00842-6 PMC8860360 35190680

[94] Chicaybam L Abdo L Viegas M Marques LVC de Sousa P Batista-Silva LR. Transposon-Mediated Generation of CAR-T Cells Shows Efficient Anti B-Cell Leukemia Response After Ex Vivo Expansion. Gene Ther (2020) 27:85–95. doi: 10.1038/s41434-020-0121-4 31919448

[95] de Macedo Abdo L Barros LRC Saldanha Viegas M Vieira Codeço Marques L de Sousa Ferreira P Chicaybam L. Development of CAR-T Cell Therapy for B-ALL Using a Point-of-Care Approach. Oncoimmunology (2020) 9:1752592. doi: 10.1080/2162402X.2020.1752592 32363126 PMC7185214

[96] Chan T Gallagher J Cheng N-L Carvajal-Borda F Plummer J Govekung A. CD19-Specific Chimeric Antigen Receptor-Modified T Cells With Safety Switch Produced Under “Point-of-Care” Using the Sleeping Beauty System for the Very Rapid Manufacture and Treatment of B-Cell Malignancies. Blood (2017) 130:1324. doi: 10.1182/blood.V130.Suppl_1.1324.1324

[97] Chan T Ma X Carvajal-Borda F Velez J Plummer J Shepard L. Preclinical Characterization of Prgn-3006 Ultracar-T^TM^ for the Treatment of AML and MDS: Non-Viral, Multigenic Autologous CAR-T Cells Administered One Day After Gene Transfer. Blood (2019) 134:2660. doi: 10.1182/blood-2019-130617

[98] Chan T Scott SP Du M Bolinger C Poortman C Shepard L. Preclinical Evaluation of Prgn-3007, a Non-Viral, Multigenic, Autologous ROR1 Ultracar-T ® Therapy With Novel Mechanism of Intrinsic PD-1 Blockade for Treatment of Hematological and Solid Cancers. Blood (2021) 138:1694. doi: 10.1182/blood-2021-149203

[99] Magnani CF Myburgh R Russkamp NF Pascolo S Shizuru JA Neri D. Anti-CD117 CAR T Cells Incorporating a Safety Switch Eradicate Acute Myeloid Leukemia and Deplete Human Hematopoietic Stem Cells. Blood (2021) 138:2808. doi: 10.1182/blood-2021-145195

[100] Biondi M Cerina B Tomasoni C Dotti G Tettamanti S Biondi A. Combining the Expression of CD33.CAR and CXCR4 to Increase CAR-CIK Cell Homing to Bone Marrow Niche and Leukemic Stem Cell Eradication in Acute Myeloid Leukemia. Blood (2021) 138:2791. doi: 10.1182/blood-2021-152394

[101] Bexte T Botezatu L Miskey C Campe J Reindl LM Gebel V. Non-Viral Sleeping Beauty Transposon Engineered CD19-CAR-NK Cells Show a Safe Genomic Integration Profile and High Antileukemic Efficiency. Blood (2021) 138:2797. doi: 10.1182/blood-2021-153999

[102] Caruso HG Tanaka R Liang J Ling X Sabbagh A Henry VK. Shortened Ex Vivo Manufacturing Time of EGFRvIII-Specific Chimeric Antigen Receptor (CAR) T Cells Reduces Immune Exhaustion and Enhances Antiglioma Therapeutic Function. J Neurooncol (2019) 145:429–39. doi: 10.1007/s11060-019-03311-y 31686330

[103] Kebriaei P Singh H Huls MH Figliola MJ Bassett R Olivares S. Phase I Trials Using Sleeping Beauty to Generate CD19-Specific CAR T Cells. J Clin Invest (2016) 126:3363–76. doi: 10.1172/JCI86721 PMC5004935 27482888

[104] Kebriaei P Huls H Olivares S Orozco AF Su S Maiti SN. Long Term Follow Up After Adoptive Transfer of CD19-Specific CAR+ T Cells Genetically Modified *Via* Non-Viral Sleeping Beauty S Ystem Following Hematopoietic Stem Cell Transplantation (HSCT). Blood (2017) 130:2059. doi: 10.1182/blood.V130.Suppl_1.2059.2059

[105] Kebriaei P Izsvák Z Narayanavari SA Singh H Ivics Z. Gene Therapy With the Sleeping Beauty Transposon System. Trends Genet (2017) 33:852–70. doi: 10.1016/j.tig.2017.08.008 28964527

[106] Magnani CF Gaipa G Lussana F Gritti G Belotti D Napolitano S. Donor-Derived CAR T Cells Engineered With Sleeping Beauty in Pediatric and Adult Patients With Acute Lymphoblastic Leukemia Relapsed Post-HSCT. Blood (2021) 138:472. doi: 10.1182/blood-2021-148703

[107] Sallman DA Elmariah H Sweet K Talati C Mishra A Cox CA. Phase 1/1b Safety Study of Prgn-3006 Ultracar-T in Patients With Relapsed or Refractory CD33-Positive Acute Myeloid Leukemia and Higher Risk Myelodysplastic Syndromes. Blood (2021) 138:825. doi: 10.1182/blood-2021-152692

[108] Chan T Chakiath M Shepard L Metenou S Carvajal-Borda F Velez J. Abstract 6593: PRGN-3005 UltraCAR-T^TM^: Multigenic CAR-T Cells Generated Using non-Viral Gene Delivery and Rapid Manufacturing Process for the Treatment of Ovarian Cancer. Cancer Res (2020) 80:6593. doi: 10.1158/1538-7445.AM2020-6593

[109] Prommersberger S Reiser M Beckmann J Danhof S Amberger M Quade-Lyssy P. CARAMBA: A First-in-Human Clinical Trial With SLAMF7 CAR-T Cells Prepared by Virus-Free Sleeping Beauty Gene Transfer to Treat Multiple Myeloma. Gene Ther (2021) 28:560–71. doi: 10.1038/s41434-021-00254-w PMC8455317 33846552

[110] Chen Q Luo W Veach RA Hickman AB Wilson MH Dyda F. Structural Basis of Seamless Excision and Specific Targeting by Piggybac Transposase. Nat Commun (2020) 11:3446. doi: 10.1038/s41467-020-17128-1 32651359 PMC7351741

[111] Bishop DC Xu N Tse B O’Brien TA Gottlieb DJ Dolnikov A. PiggyBac-Engineered T Cells Expressing CD19-Specific CARs That Lack IgG1 Fc Spacers Have Potent Activity Against B-ALL Xenografts. Mol Ther (2018) 26:1883–95. doi: 10.1016/j.ymthe.2018.05.007 PMC6094355 29861327

[112] Yusa K Zhou L Li MA Bradley A Craig NL. A Hyperactive Piggybac Transposase for Mammalian Applications. Proc Natl Acad Sci USA (2011) 108:1531–6. doi: 10.1073/pnas.1008322108 PMC3029773 21205896

[113] Li X Burnight ER Cooney AL Malani N Brady T Sander JD. PiggyBac Transposase Tools for Genome Engineering. Proc Natl Acad Sci USA (2013) 110(25):e2279–e2287. doi: 10.1073/pnas.1305987110 23723351 PMC3690869

[114] Liu G Aronovich EL Cui Z Whitley CB Hackett PB. Excision of Sleeping Beauty Transposons: Parameters and Applications to Gene Therapy. J Gene Med (2004) 6(5):574–83. 10.1002/jgm.486 PMC1865527 15133768

[115] Chiung-Yuan Wu S James Meir Y-J Coates CJ Handler AM Pelczar P Moisyadi S. Piggybac is a Flexible and Highly Active Transposon as Compared to Sleeping Beauty, Tol2, and Mos1 in Mammalian Cells. Natl Acad Sci USA (2006) 103(41):15008–13. doi: 10.1073/pnas.0606979103 PMC1622771 17005721

[116] Zhao S Jiang E Chen S Gu Y Shangguan AJ Lv T. PiggyBac Transposon Vectors: The Tools of the Human Gene Encoding. Transl Lung Cancer Res (2016) 5:120–5. doi: 10.3978/j.issn.2218-6751.2016.01.05 PMC4758974 26958506

[117] de Jong J Akhtar W Badhai J Rust AG Rad R Hilkens J. Chromatin Landscapes of Retroviral and Transposon Integration Profiles. PloS Genet (2014) 10:e1004250. doi: 10.1371/journal.pgen.1004250 24721906 PMC3983033

[118] Henssen AG Koche R Zhuang J Jiang E Reed C Eisenberg A. PGBD5 Promotes Site-Specific Oncogenic Mutations in Human Tumors. Nat Genet (2017) 49:1005–14. doi: 10.1038/ng.3866 PMC5489359 28504702

[119] Beckermann TM Luo W Wilson CM Veach RA Wilson MH. Cognate Restriction of Transposition by Piggybac-Like Proteins. Nucleic Acids Res (2021) 49:8135–44. doi: 10.1093/nar/gkab578 PMC8373079 34232995

[120] Nakazawa Y Huye LE Dotti G Foster AE Vera JF Manuri PR. Optimization of the PiggyBac Transposon System for the Sustained Genetic Modification of Human T Lymphocytes. J Immunother (2009) 32(8):826–36. doi: 10.1097/CJI.0b013e3181ad762b PMC2796278 19752751

[121] Morita D Nishio N Saito S Tanaka M Kawashima N Okuno Y. Enhanced Expression of Anti-CD19 Chimeric Antigen Receptor in Piggybac Transposon-Engineered T Cells. Mol Ther - Methods Clin Dev (2018) 8:131–40. doi: 10.1016/j.omtm.2017.12.003 PMC5907825 29687032

[122] Tanaka K Kato I Tanaka M Morita D Matsuda K Takahashi Y. Direct Delivery of Piggybac CD19 CAR T Cells Has Potent Anti-Tumor Activity Against ALL Cells in CNS in a Xenograft Mouse Model. Mol Ther - Oncolytics (2020) 18:37–46. doi: 10.1016/j.omto.2020.05.013 32637579 PMC7321814

[123] Biagi E Biondi A Magnani CF Tettamanti S. Improved Method for the Generation of Genetically Modified Cells. Eur patent EP3018200A1 (2014).

[124] Ramanayake S Bilmon I Bishop D Dubosq M-C Blyth E Clancy L. Low-Cost Generation of Good Manufacturing Practice–Grade CD19-Specific Chimeric Antigen Receptor–Expressing T Cells Using Piggybac Gene Transfer and Patient-Derived Materials. Cytotherapy (2015) 17:1251–67. doi: 10.1016/j.jcyt.2015.05.013 26212611

[125] Kaštánková I Štach M Žižková H Ptáčková P Šmilauerová K Mucha M. Enzymatically Produced Piggybac Transposon Vectors for Efficient non-Viral Manufacturing of CD19-Specific CAR T Cells. Mol Ther - Methods Clin Dev (2021) 23:119–27. doi: 10.1016/j.omtm.2021.08.006 PMC8482285 34631931

[126] Nakamura K Yagyu S Hirota S Tomida A Kondo M Shigeura T. Autologous Antigen-Presenting Cells Efficiently Expand Piggybac Transposon CAR-T Cells With Predominant Memory Phenotype. Mol Ther - Methods Clin Dev (2021) 21:315–24. doi: 10.1016/j.omtm.2021.03.011 PMC8047430 33898630

[127] Tomida A Yagyu S Nakamura K Kubo H Yamashima K Nakazawa Y. Inhibition of MEK Pathway Enhances the Antitumor Efficacy of Chimeric Antigen Receptor T Cells Against Neuroblastoma. Cancer Sci (2021) 112:4026–36. doi: 10.1111/cas.15074 PMC8486218 34382720

[128] Morokawa H Yagyu S Hasegawa A Tanaka M Saito S Mochizuki H. Autologous Non-Human Primate Model for Safety Assessment of Piggybac Transposon-Mediated Chimeric Antigen Receptor T Cells on Granulocyte–Macrophage Colony-Stimulating Factor Receptor. Clin Transl Immunol (2020) 9(11):e1207. doi: 10.1002/cti2.1207 PMC7680920 33251009

[129] Hasegawa A Saito S Narimatsu S Nakano S Nagai M Ohnota H. Mutated GM-CSF-Based CAR-T Cells Targeting CD116/CD131 Complexes Exhibit Enhanced Anti-Tumor Effects Against Acute Myeloid Leukaemia. Clin Transl Immunol (2021) 10(5):e1282. doi: 10.1002/cti2.1282 PMC8102137 33976880

[130] Bishop DC Caproni L Gowrishankar K Legiewicz M Karbowniczek K Tite J. CAR T Cell Generation by Piggybac Transposition From Linear Doggybone DNA Vectors Requires Transposon DNA-Flanking Regions. Mol Ther - Methods Clin Dev (2020) 17:359–68. doi: 10.1016/j.omtm.2019.12.020 PMC7016334 32071928

[131] Ptáčková P Musil J Štach M Lesný P Němečková Š Král V. A New Approach to CAR T-Cell Gene Engineering and Cultivation Using Piggybac Transposon in the Presence of IL-4, IL-7 and IL-21. Cytotherapy (2018) 20:507–20. doi: 10.1016/j.jcyt.2017.10.001 29475789

[132] Micklethwaite KP Gowrishankar K Gloss BS Li Z Street JA Moezzi L. Investigation of Product-Derived Lymphoma Following Infusion of Piggybac-Modified CD19 Chimeric Antigen Receptor T Cells. Blood (2021) 138:1391–405. doi: 10.1182/blood.2021010858 PMC8532197 33974080

[133] Daniels J Choi J. BACH2 is a Putative T-Cell Lymphoma Tumor Suppressor That may Play a Role in Product-Derived CAR T-Cell Lymphomas. Blood (2021) 138:2731–3. doi: 10.1182/blood.2021012641 PMC8703361 34499707

[134] Cesana D Santoni de Sio FR Rudilosso L Gallina P Calabria A Beretta S. HIV-1-Mediated Insertional Activation of STAT5B and BACH2 Trigger Viral Reservoir in T Regulatory Cells. Nat Commun (2017) 8:498. doi: 10.1038/s41467-017-00609-1 28887441 PMC5591266

[135] Nishio N Hanajiri R Ishikawa Y Murata M Taniguchi R Hamada M. A Phase I Study of CD19 Chimeric Antigen Receptor-T Cells Generated By the PiggyBac Transposon Vector for Acute Lymphoblastic Leukemia. Blood (2021) 138:3831. doi: 10.1182/blood-2021-150469

[136] Zhang Y Zhang Z Ding Y Fang Y Wang P Chu W. Phase I Clinical Trial of EGFR-Specific CAR-T Cells Generated by the Piggybac Transposon System in Advanced Relapsed/Refractory non-Small Cell Lung Cancer Patients. J Cancer Res Clin Oncol (2021) 147:3725–34. doi: 10.1007/s00432-021-03613-7 PMC11801842 34032893

[137] Costello C Derman BA Kocoglu MH Deol A Ali AA Gregory T. Clinical Trials of BCMA-Targeted CAR-T Cells Utilizing a Novel Non-Viral Transposon System. Blood (2021) 138:3858. doi: 10.1182/blood-2021-151672

[138] Dolgin E. The Tangled History of mRNA Vaccines. Nature (2021) 597:318–24. doi: 10.1038/d41586-021-02483-w 34522017

[139] Kuhn AN Diken M Kreiter S Vallazza B Türeci Ö Sahin U. Determinants of Intracellular RNA Pharmacokinetics: Implications for RNA-Based Immunotherapeutics. RNA Biol (2011) 8:35–43. doi: 10.4161/rna.8.1.13767 21289486

[140] Malone RW Felgner PL Verma IM. Cationic Liposome-Mediated RNA Transfection. Proc Natl Acad Sci USA (1989) 86:6077–81. doi: 10.1073/pnas.86.16.6077 PMC297778 2762315

[141] Simon B Harrer DC Schuler-Thurner B Schaft N Schuler G Dörrie J. The siRNA-Mediated Downregulation of PD-1 Alone or Simultaneously With CTLA-4 Shows Enhanced *In Vitro* CAR-T-Cell Functionality for Further Clinical Development Towards the Potential Use in Immunotherapy of Melanoma. Exp Dermatol (2018) 27:769–78. doi: 10.1111/exd.13678 29704887

[142] Siddiqi T Soumerai JD Wierda WG Dubovsky JA Gillenwater HH Gong L. Rapid MRD-Negative Responses in Patients With Relapsed/Refractory CLL Treated With Liso-Cel, a CD19-Directed CAR T-Cell Product: Preliminary Results From Transcend CLL 004, a Phase 1/2 Study Including Patients With High-Risk Disease Previously Treated With Ibrutinib. Blood (2018) 132:300. doi: 10.1182/blood-2018-99-110462

[143] Granot-Matok Y Kon E Dammes N Mechtinger G Peer D. Therapeutic mRNA Delivery to Leukocytes. J Control Release (2019) 305:165–75. doi: 10.1016/j.jconrel.2019.05.032 31121277

[144] Şen A Kargar K Akgün E Pınar MÇ. Codon Optimization: A Mathematical Programing Approach. Bioinformatics (2020) 36:4012–20. doi: 10.1093/bioinformatics/btaa248 32311016

[145] Tcherepanova IY Adams MD Feng X Hinohara A Horvatinovich J Calderhead D. Ectopic Expression of a Truncated CD40L Protein From Synthetic Post-Transcriptionally Capped RNA in Dendritic Cells Induces High Levels of IL-12 Secretion. BMC Mol Biol (2008) 9:90. doi: 10.1186/1471-2199-9-90 18928538 PMC2576345

[146] Tcherepanova I Harris J Starr A Cleveland J Ketteringham H Calderhead D. Multiplex RT-PCR Amplification of HIV Genes to Create a Completely Autologous DC-Based Immunotherapy for the Treatment of HIV Infection. PloS One (2008) 3:e1489. doi: 10.1371/journal.pone.0001489 18231576 PMC2211536

[147] Karikó K Muramatsu H Welsh FA Ludwig J Kato H Akira S. Incorporation of Pseudouridine Into mRNA Yields Superior Nonimmunogenic Vector With Increased Translational Capacity and Biological Stability. Mol Ther (2008) 16:1833–40. doi: 10.1038/mt.2008.200 PMC2775451 18797453

[148] Jackson LA Anderson EJ Rouphael NG Roberts PC Makhene M Coler RN. An mRNA Vaccine Against SARS-CoV-2 - Preliminary Report. N Engl J Med (2020) 383:1920–31. doi: 10.1056/NEJMoa2022483 PMC7377258 32663912

[149] Mulligan MJ Lyke KE Kitchin N Absalon J Gurtman A Lockhart S. Phase I/II Study of COVID-19 RNA Vaccine BNT162b1 in Adults. Nature (2020) 586:589–93. doi: 10.1038/s41586-020-2639-4 32785213

[150] Pasquinelli AE Dahlberg JE Lund E. Reverse 5’ Caps in RNAs Made *In Vitro* by Phage RNA Polymerases. RNA (1995) 1:957–67.PMID 8548660 PMC1369344 8548660

[151] Stepinski J Waddell C Stolarski R Darzynkiewicz E Rhoads RE. Synthesis and Properties of mRNAs Containing the Novel “Anti-Reverse” Cap Analogs 7-Methyl(3’-O-Methyl)GpppG and 7-Methyl (3’-Deoxy)GpppG. RNA (2001) 7:1486–95. PMC1370192 11680853

[152] Venkatesan S Gershowitz A Moss B. Modification of the 5’ End of mRNA. Association of RNA Triphosphatase With the RNA Guanylyltransferase-RNA (Guanine-7-)Methyltransferase Complex From Vaccinia Virus. J Biol Chem (1980) 255:903–8. doi: 10.1016/S0021-9258(19)86118-5 6243301

[153] Kuge H Brownlee GG Gershon PD Richter JD. Cap Ribose Methylation of C-Mos mRNA Stimulates Translation and Oocyte Maturation in Xenopus Laevis. Nucleic Acids Res (1998) 26:3208–14. doi: 10.1093/nar/26.13.3208 PMC147664 9628920

[154] Elango N Elango S Shivshankar P Katz MS. Optimized Transfection of mRNA Transcribed From a D(A/T)100 Tail-Containing Vector. Biochem Biophys Res Commun (2005) 330:958–66. doi: 10.1016/j.bbrc.2005.03.067 15809089

[155] Holtkamp S Kreiter S Selmi A Simon P Koslowski M Huber C. Modification of Antigen-Encoding RNA Increases Stability, Translational Efficacy, and T-Cell Stimulatory Capacity of Dendritic Cells. Blood (2006) 108:4009–17. doi: 10.1182/blood-2006-04-015024 16940422

[156] Gingras AC Raught B Sonenberg N. Eif4 Initiation Factors: Effectors of mRNA Recruitment to Ribosomes and Regulators Of Translation. Annu Rev Biochem (1999) 68:913–63. doi: 10.1146/annurev.biochem.68.1.913 10872469

[157] Holcik M Liebhaber SA. Four Highly Stable Eukaryotic mRNAs Assemble 3’ Untranslated Region RNA-Protein Complexes Sharing Cis and Trans Components. Proc Natl Acad Sci USA (1997) 94:2410–4. doi: 10.1073/pnas.94.6.2410 PMC20101 9122208

[158] van der Velden AW Thomas AA. The Role of the 5’ Untranslated Region of an mRNA in Translation Regulation During Development. Int J Biochem Cell Biol (1999) 31:87–106. doi: 10.1016/s1357-2725(98)00134-4 10216946

[159] Vivinus S Baulande S van Zanten M Campbell F Topley P Ellis JH. An Element Within the 5’ Untranslated Region of Human Hsp70 mRNA Which Acts as a General Enhancer of mRNA Translation. Eur J Biochem (2001) 268:1908–17. doi: 10.1046/j.1432-1327.2001.02064.x 11277913

[160] Yu J Russell JE. Structural and Functional Analysis of an mRNP Complex That Mediates the High Stability of Human Beta-Globin mRNA. Mol Cell Biol (2001) 21:5879–88. doi: 10.1128/MCB.21.17.5879-5888.2001 PMC87307 11486027

[161] Barro M Bravo C Spencer E. Differential Usage of RNA Templates by the Rotavirus “*In Vitro*” Replication System. Arch Virol (2004) 149:1815–29. doi: 10.1007/s00705-004-0314-y 15593422

[162] Van Tendeloo VF Ponsaerts P Lardon F Nijs G Lenjou M Van Broeckhoven C. Highly Efficient Gene Delivery by mRNA Electroporation in Human Hematopoietic Cells: Superiority to Lipofection and Passive Pulsing of mRNA and to Electroporation of Plasmid cDNA for Tumor Antigen Loading of Dendritic Cells. Blood (2001) 98:49–56. doi: 10.1182/blood.v98.1.49 11418462

[163] Zohra FT Chowdhury EH Tada S Hoshiba T Akaike T. Effective Delivery With Enhanced Translational Activity Synergistically Accelerates mRNA-Based Transfection. Biochem Biophys Res Commun (2007) 358:373–8. doi: 10.1016/j.bbrc.2007.04.059 17475211

[164] Huth S Hoffmann F von Gersdorff K Laner A Reinhardt D Rosenecker J. Interaction of Polyamine Gene Vectors With RNA Leads to the Dissociation of Plasmid DNA-Carrier Complexes. J Gene Med (2006) 8:1416–24. doi: 10.1002/jgm.975 17029296

[165] Soundara Rajan T Gugliandolo A Bramanti P Mazzon E. *In Vitro-*Transcribed mRNA Chimeric Antigen Receptor T Cell (IVT mRNA CAR T) Therapy in Hematologic and Solid Tumor Management: A Preclinical Update. Int J Mol Sci (2020) 21(18): 6514. doi: 10.3390/ijms21186514 PMC7556036 32899932

[166] Beatty GL Haas AR Maus MV Torigian DA Soulen MC Plesa G. Mesothelin-Specific Chimeric Antigen Receptor mRNA-Engineered T Cells Induce Anti-Tumor Activity in Solid Malignancies. Cancer Immunol Res (2014) 2:112–20. doi: 10.1158/2326-6066.CIR-13-0170 PMC3932715 24579088

[167] Zhao Y Moon E Carpenito C Paulos CM Liu X Brennan AL. Multiple Injections of Electroporated Autologous T Cells Expressing a Chimeric Antigen Receptor Mediate Regression of Human Disseminated Tumor. Cancer Res (2010) 70:9053–61. doi: 10.1158/0008-5472.CAN-10-2880 PMC2982929 20926399

[168] Wilber A Frandsen JL Geurts JL Largaespada DA Hackett PB McIvor RS. RNA as a Source of Transposase for Sleeping Beauty-Mediated Gene Insertion and Expression in Somatic Cells and Tissues. Mol Ther (2006) 13:625–30. doi: 10.1016/j.ymthe.2005.10.014 16368272

[169] Wilber A Wangensteen KJ Chen Y Zhuo L Frandsen JL Bell JB. Messenger RNA as a Source of Transposase for Sleeping Beauty Transposon-Mediated Correction of Hereditary Tyrosinemia Type I. Mol Ther (2007) 15:1280–7. doi: 10.1038/sj.mt.6300160 17440442

[170] Bire S Ley D Casteret S Mermod N Bigot Y Rouleux-Bonnin F. Optimization of the Piggybac Transposon Using mRNA and Insulators: Toward a More Reliable Gene Delivery System. PloS One (2013) 8:e82559. doi: 10.1371/journal.pone.0082559 24312663 PMC3849487

[171] Liang Q Kong J Stalker J Bradley A. Chromosomal Mobilization and Reintegration of Sleeping Beauty and PiggyBac Transposons. Genesis (2009) 47:404–8. doi: 10.1002/dvg.20508 19391106

[172] Galla M Schambach A Falk CS Maetzig T Kuehle J Lange K. Avoiding Cytotoxicity of Transposases by Dose-Controlled mRNA Delivery. Nucleic Acids Res (2011) 39:7147–60. doi: 10.1093/nar/gkr384 PMC3167617 21609958

[173] Maus MV Haas AR Beatty GL Albelda SM Levine BL Liu X. T Cells Expressing Chimeric Antigen Receptors can Cause Anaphylaxis in Humans. Cancer Immunol Res (2013) 1:26–31. doi: 10.1158/2326-6066.CIR-13-0006 PMC3888798 24777247

[174] Cummins KD Frey N Nelson AM Schmidt A Luger S Isaacs RE. Treating Relapsed / Refractory (RR) AML With Biodegradable Anti-CD123 CAR Modified T Cells. Blood (2017) 130:1359. doi: 10.1182/blood.V130.Suppl_1.1359.1359

[175] Smith RP Riordan JD Feddersen CR Dupuy AJ. A Hybrid Adenoviral Vector System Achieves Efficient Long-Term Gene Expression in the Liver *via* Piggybac Transposition. Hum Gene Ther (2015) 26:377–85. doi: 10.1089/hum.2014.123 PMC4492551 25808258

[176] La QT Ren B Logan GJ Cunningham SC Khandekar N Nassif NT. Use of a Hybrid Adeno-Associated Viral Vector Transposon System to Deliver the Insulin Gene to Diabetic NOD Mice. Cells (2020) 9(10):2227. doi: 10.3390/cells9102227 PMC7600325 33023100

[177] Cooney AL Thornell IM Singh BK Shah VS Stoltz DA McCray PBJ. A Novel AAV-Mediated Gene Delivery System Corrects CFTR Function in Pigs. Am J Respir Cell Mol Biol (2019) 61:747–54. doi: 10.1165/rcmb.2019-0006OC PMC6890402 31184507

[178] Boehme P Zhang W Solanki M Ehrke-Schulz E Ehrhardt A. A High-Capacity Adenoviral Hybrid Vector System Utilizing the Hyperactive Sleeping Beauty Transposase SB100X for Enhanced Integration. Mol Ther Nucleic Acids (2016) 5:e337. doi: 10.1038/mtna.2016.44 27434682 PMC5330939

[179] Hudecek M Izsvák Z Johnen S Renner M Thumann G Ivics Z. Going non-Viral: The Sleeping Beauty Transposon System Breaks on Through to the Clinical Side. Crit Rev Biochem Mol Biol (2017) 52:355–80. doi: 10.1080/10409238.2017.1304354 28402189

[180] Smith TT Stephan SB Moffett HF McKnight LE Ji W Reiman D. *In Situ* Programming of Leukaemia-Specific T Cells Using Synthetic DNA Nanocarriers. Nat Nanotechnol (2017) 12:813–22. doi: 10.1038/NNANO.2017.57 PMC5646367 28416815

[181] Parayath NN Stephan SB Koehne AL Nelson PS Stephan MT. *In Vitro*-Transcribed Antigen Receptor mRNA Nanocarriers for Transient Expression in Circulating T Cells. *In Vivo* . Nat Commun (2020) 11:6080. doi: 10.1038/s41467-020-19486-2 33247092 PMC7695830

[182] Rurik JG Tombacz I Y A O. MF P Shewale SV Li L. CAR T Cells Produced *In Vivo* to Treat Cardiac Injury. Science (80-) (2022) 375:91–6. doi: 10.1126/science.abm0594 PMC9983611 34990237

[183] Gong N Sheppard NC Billingsley MM June CH Mitchell MJ. Nanomaterials for T-Cell Cancer Immunotherapy. Nat Nanotechnol (2021) 16:25–36. doi: 10.1038/s41565-020-00822-y 33437036

[184] Bozza M De Roia A Correia MP Berger A Tuch A Schmidt A. APPLIED SCIENCES AND ENGINEERING A Nonviral, Nonintegrating DNA Nanovector Platform for the Safe, Rapid, and Persistent Manufacture of Recombinant T Cells. Sci Adv (2021) 7(16):eabf1333. doi: 10.1126/sciadv.abf1333 33853779 PMC8046366

[185] Cardle II Cheng EL Jensen MC Pun SH. Biomaterials in Chimeric Antigen Receptor T-Cell Process Development. Acc Chem Res (2020) 53:1724–38. doi: 10.1021/acs.accounts.0c00335 32786336

[186] Abou-El-Enein M Elsallab M Feldman SA Fesnak AD Heslop HE Marks P. Scalable Manufacturing of CAR T Cells for Cancer Immunotherapy. Blood Cancer Discov (2021) 2:408–22. doi: 10.1158/2643-3230.BCD-21-0084 PMC8462122 34568831

[187] Stadtmauer EA Fraietta JA Davis MM Cohen AD Weber KL Lancaster E. CRISPR-Engineered T Cells in Patients With Refractory Cancer. Science (2020) 367(6481):eaba7365. doi: 10.1126/science.aba7365 32029687 PMC11249135

[188] Roth TL Puig-Saus C Yu R Shifrut E Carnevale J Li PJ. Reprogramming Human T Cell Function and Specificity With Non-Viral Genome Targeting. Nature (2018) 559:405–9. doi: 10.1038/s41586-018-0326-5 PMC6239417 29995861

[189] Schober K Müller TR Gökmen F Grassmann S Effenberger M Poltorak M. Orthotopic Replacement of T-Cell Receptor α- and β-Chains With Preservation of Near-Physiological T-Cell Function. Nat BioMed Eng (2019) 3:974–84. doi: 10.1038/s41551-019-0409-0 31182835

[190] Müller TR Jarosch S Hammel M Leube J Grassmann S Bernard B. Targeted T Cell Receptor Gene Editing Provides Predictable T Cell Product Function for Immunotherapy. Cell Rep Med (2021) 2(8):100374. doi: 10.1016/j.xcrm.2021.100374 34467251 PMC8385324

[191] Nguyen DN Roth TL Li PJ Chen PA Apathy R Mamedov MR. Polymer-Stabilized Cas9 Nanoparticles and Modified Repair Templates Increase Genome Editing Efficiency. Nat Biotechnol (2020) 38:44–9. doi: 10.1038/s41587-019-0325-6 PMC6954310 31819258

[192] Kath J Du W Thommandru B Turk R Amini L Stein M. Fast, Efficient and Virus-Free Generation of &Lt;Em<TRAC&lt;/em<-Replaced CAR T Cells. bioRxiv (2021) 2021.02.14.431017. doi: 10.1101/2021.02.14.431017

[193] Mueller KP Piscopo NJ Forsberg MH Saraspe LA Das A Russell B. Production and Characterization of Virus-Free, CRISPR-CAR T Cells Capable of Inducing Solid Tumor Regression. bioRxiv (2021) 2021.08.06.455489. doi: 10.1101/2021.08.06.455489 PMC9454086 36382633

[194] Shy BR Vykunta V Ha A Roth TL Talbot A Nguyen DN. Hybrid ssDNA Repair Templates Enable High Yield Genome Engineering in Primary Cells for Disease Modeling and Cell Therapy Manufacturing. bioRxiv (2021) 2021.09.02.458799. doi: 10.1101/2021.09.02.458799

[195] Strecker J Ladha A Gardner Z Schmid-Burgk JL Makarova KS Koonin EV. RNA-Guided DNA Insertion With CRISPR-Associated Transposases. Science (2019) 365:48–53. doi: 10.1126/science.aax9181 31171706 PMC6659118

[196] Klompe SE Vo PLH Halpin-Healy TS Sternberg SH. Transposon-Encoded CRISPR–Cas Systems Direct RNA-Guided DNA Integration. Nature (2019) 571:219–25. doi: 10.1038/s41586-019-1323-z 31189177

[197] Kovač A Miskey C Menzel M Grueso E Gogol-Döring A Ivics Z. RNA-Guided Retargeting of Sleeping Beauty Transposition in Human Cells. Elife (2020) 9:e53868. doi: 10.7554/eLife.53868 32142408 PMC7077980

